# Diagnosing ventilator-associated pneumonia (VAP) in UK NHS ICUs: the perceived value and role of a novel optical technology

**DOI:** 10.1186/s41512-022-00117-x

**Published:** 2022-02-10

**Authors:** W. S. Jones, J. Suklan, A. Winter, K. Green, T. Craven, A. Bruce, J. Mair, K. Dhaliwal, T. Walsh, A. J. Simpson, S. Graziadio, A. J. Allen

**Affiliations:** 1 NIHR Newcastle In Vitro Diagnostics Co-operative, Newcastle upon Tyne Hospitals Foundation Trust, Newcastle upon Tyne, NE1 4LP UK; 2 NIHR Newcastle In Vitro Diagnostics Co-operative, Translational & Clinical Research Institute, Newcastle University, Newcastle upon Tyne, NE2 4HH UK; 3 Translational Healthcare Technologies Group, Queen’s Medical Research Institute, University of Edinburgh, Edinburgh, EH16 4TJ UK; 4 Edinburgh Critical Care Research Group, University of Edinburgh, Edinburgh, UK

**Keywords:** Ventilator-associated pneumonia, VAP, Interviews, Care pathway analysis, Thematic analysis

## Abstract

**Background:**

Diagnosing ventilator-associated pneumonia (VAP) in an intensive care unit (ICU) is a complex process. Our aim was to collect, evaluate and represent the information relating to current clinical practice for the diagnosis of VAP in UK NHS ICUs, and to explore the potential value and role of a novel diagnostic for VAP, which uses optical molecular alveoscopy to visualise the alveolar space.

**Methods:**

Qualitative study performing semi-structured interviews with clinical experts. Interviews were recorded, transcribed, and thematically analysed. A flow diagram of the VAP patient pathway was elicited and validated with the expert interviewees. Fourteen clinicians were interviewed from a range of UK NHS hospitals: 12 ICU consultants, 1 professor of respiratory medicine and 1 professor of critical care.

**Results:**

Five themes were identified, relating to [1] current practice for the diagnosis of VAP, [2] current clinical need in VAP diagnostics, [3] the potential value and role of the technology, [4] the barriers to adoption and [5] the evidence requirements for the technology, to help facilitate a successful adoption. These themes indicated that diagnosis of VAP is extremely difficult, as is the decision to stop antibiotic treatment. The analysis revealed that there is a clinical need for a diagnostic that provides an accurate and timely diagnosis of the causative pathogen, without the long delays associated with return of culture results, and which is not dangerous to the patient. It was determined that the technology would satisfy important aspects of this clinical need for diagnosing VAP (and pneumonia, more generally), but would require further evidence on safety and efficacy in the patient population to facilitate adoption.

**Conclusions:**

Care pathway analysis performed in this study was deemed accurate and representative of current practice for diagnosing VAP in a UK ICU as determined by relevant clinical experts, and explored the value and role of a novel diagnostic, which uses optical technology, and could streamline the diagnostic pathway for VAP and other pneumonias.

**Supplementary Information:**

The online version contains supplementary material available at 10.1186/s41512-022-00117-x.

## Introduction

Pneumonia is a bacterial, viral or fungal infection of the lungs, which causes the alveoli of the lungs to fill up with microorganisms, fluid and inflammatory cells, preventing the lungs from functioning effectively [1]. The classification scheme for pneumonia in UK NHS hospitals is based on the setting in which the infection was mostly likely acquired: community-acquired pneumonia (CAP), when a patient is in the hospital <48h before the pneumonia is suspected; hospital-acquired pneumonia (HAP), when a patient is in the hospital >48h before the pneumonia develops, but is not associated with mechanical ventilation; and ventilator-associated pneumonia (VAP), when a patient is mechanically ventilated and intubated for >48h before the pneumonia develops [2–4]. The causative pathogens in each scenario are different, and treatment strategies vary as a consequence.

Acquiring a pneumonia in the ICU can have severe consequences for the patient. For example, VAP in the ICU is the leading cause of death relating to infection [3, 5–7] and is associated with increased duration of mechanical ventilation, length of stay (in the ICU and hospital), morbidity and healthcare costs [8–11].

With the rise of novel coronavirus SARS-CoV-2 and the ensuing global pandemic, many countries have reported a sharp rise in ventilator use in ICU, prompting governments and health services to order vast quantities of ventilators to meet the growing demand. In the UK, initial estimates of ventilators required to deal with this influx stood at 30,000 in early March 2019 before being revised in April 2019 to 18,000, still 10,000 units more than were in use in UK NHS practices at the start of that year [12]. A report from the Intensive Care National Audit described two-thirds of COVID-19 patients in the UK requiring critical care were put on mechanical ventilation within 24 h of admission with a median length of stay of 3–5 days depending on the level of support required [13]. The increased use of ventilators, driven in part by the spread of COVID-19, will likely have an ongoing impact on the number of reported cases of VAP and bring about greater pressure for accurate and timely diagnosis of VAP.

**Fig. 1:**
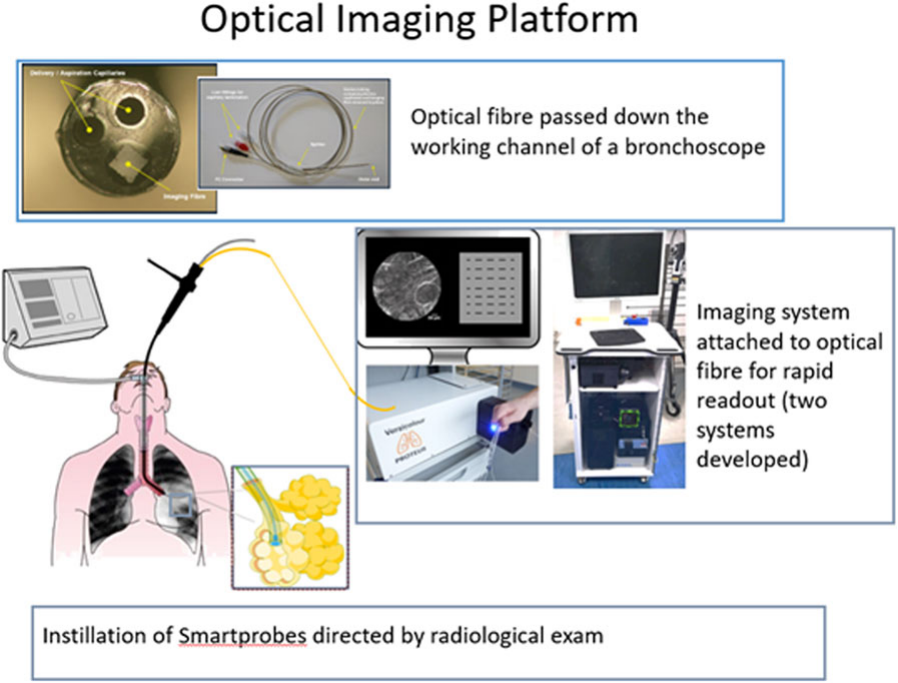
Image showing a bronchoscopy procedure with the imaging fibre and capillary bundle being passed down the working channel of a bronchoscope into the alveolar regions. SmartProbes are delivered via the capillaries and images are sent via the imaging fibre to the imaging system at the patient’s bedside

The exponential growth in scientific and technological advancement has led to the development of several novel devices for the diagnosis of infections (in general) and pneumonia (in particular) [14–22]. The Translational Healthcare Technologies group [23] have developed an optical molecular alveoscopy (OMA) platform for the potential diagnosis of pneumonia at the bedside in the ICU setting (Fig. 1)*.* The OMA platform administers SmartProbes (microdoses of optical molecular imaging reagents that are delivered into the distal lung) to detect infection and inflammation, in real time. These molecules fluoresce/light up when they bind to specific targets such as bacteria or activated neutrophils. Current clinically developed SmartProbes are specific for some gram-negative bacteria, gram-positive bacteria and/or neutrophils. These Smartprobes are delivered through a multi-functional bundle (Panoptes fibre) that has been extended through serial transbronchial passes into the alveolar sacs, via the working channel of a bronchoscope. Panoptes is a triple lumen optical imaging, delivery and sampling device comprised of two delivery/aspiration capillaries and an imaging fibre. Two imaging systems (Versicolour and Kronoscan) support the real-time visualisation of fluorescent bacteria and activated neutrophils within the patient’s alveolar spaces. The OMA platform also has the capacity to perform a mini-lavage by extracting small volumes of liquid instilled in the alveolar space, which could be used for culturing and confirmation of infection.

The accuracy and utility of a diagnostic test is not a fixed property, but is dependent on the clinical setting and patient population in which it is used [24]. To establish the accuracy and clinical utility of a diagnostic device, it is first necessary to understand the current diagnostic practice/pathway for the disease of interest.

Care pathway analysis, in the context of diagnostics, involves the collection, evaluation and representation of information relating to the diagnostic journey (the pathway) a patient group follows as part of a healthcare system [25–29]. In this study, we performed a set of qualitative, semi-structured interviews with clinical experts in order to conduct a care pathway analysis of the current practice for diagnosing VAP in UK NHS ICUs.

Understanding the pathway helps to determine the optimal role, setting and patient population for a new diagnostic, as well as the barriers and facilitators to adoption and future evidence requirements [27]. As part of this pathway analysis, we also explored the potential value and role of the OMA platform in the diagnosis of VAP in the ICU. Broadly speaking, there are 4 possible roles for medical tests: (1) *Screening*, to determine if asymptomatic individuals have a disease, sometimes called surveillance in ICUs; (2) *Diagnostic*, to determine if an individual has a disease; (3) *Prognostic*, to predict the likelihood of an individual developing a disease or deteriorating; and (4) *Monitoring*, to determine whether a patient’s disease is controlled or is responding to treatment. Depending on how a test will be used, it may also be further categorised as a rule-in or a rule-out test [27]. A rule-in test typically requires high specificity (low false-positive rate), so that most non-diseased subjects will be diagnosed as non-diseased. Therefore, a positive result makes the presence of disease likely, effectively ruling-in disease. A rule-out test typically requires high sensitivity, so that most diseased subjects will be diagnosed as diseased. Therefore, a negative result essentially rules out the disease in question. Rule-in tests are important when confirming a diagnosis following other clinical data or when subsequent tests or treatments are dangerous to the patient. Rule-out tests are important when there are severe consequences for missing a disease.

Recently, Korevaar et al. [24] have recommended that in evaluating a medical test one consider where it would be placed, and how it will affect the current pathway: whether it is a *triage test*, with the results determining which patients will undergo the existing test(s); an *add-on test*, used before or after an existing test(s) to improve accuracy; a *replacement test*, replacing the existing test(s), expected to be more accurate, less invasive, less costly or more usable than the test replaced; and a *new test*, where a completely new test is added to the pathway, where there was not one previously. This consideration will be referred to as its ‘role in the pathway’, to distinguish it from its ‘role as a medical test’, described in the previous paragraph. In this study, we sought to elicit information on the optimal role (in both senses) of the OMA platform for use with suspected VAP patients in UK NHS ICUs.

## Methods

### Interview structure

Semi-structured interviews were conducted with 14 clinicians from a range of UK NHS hospitals: 12 ICU consultants, 1 professor of respiratory medicine and 1 professor of critical care. Two pilot interviews were performed with local ICU consultant intensivists, to refine the interview schedule. Interviews lasted between 45 and 60 min. Interviews took place in 2019. They were a mixture of face-to-face and telephone interviews and were performed by WSJ and JS. A topic guide was developed prior to the interviews (see Supplementary Material, Appendix 1). Participants were invited to be interviewed via the UK Critical Care Research Group (UKCCRG) mailing list. The UKCCRG mailing list comprises individuals (clinicians, researchers and other key stakeholders) involved in conducting research in the critically ill in the UK. To participate in the study, the respondent was required to have significant experience of diagnosing VAP in UK ICUs. We determined this to be restricted to clinicians (consultant ICU intensivists or microbiologists) and academic clinicians with a research focus on VAP or critical care. This criterion was described in the circular email, with reminder emails used. All respondents were included in the study.

The determination of sample size used in this study was selected based on the concept of data saturation [30] and is consistent with previously published, qualitative research of this type [25].

During the interviews, participants were first asked questions on the current care pathway for the diagnosis of VAP in their ICU. Next, participants watched a short video demonstrating the OMA platform; showing the same video to all participants reduced the potential for bias. Finally, after watching the video, the participants were asked questions about the potential value and role of the OMA platform in the diagnosis of a suspected VAP in the ICU.

### Patient and public involvement

The research question and outcome measures in this study were not informed by patients’ priorities, experience and/or preferences. Patients were not involved in the study design. Patients were not involved in recruitment to, or the conduct of, this study. Patient engagement is part of the dissemination strategy. The research team plans to present the results of this study at various meetings and conferences, where patients and the public will be present.

### Data analysis

Thematic analysis was used to identify the relevant themes from the interviews [31–34]. We used the Gale et al. Framework Analysis approach [35]; see Supplementary Material, Appendix 2, for details of this approach. WSJ and JS coded and analysed the data. WSJ and JS are NIHR diagnostic test methodologists and have 8 years of combined experience in the elicitation of diagnostic pathways through interviews with clinicians and other key stakeholders, and in reaching consensus on themes.

## Results

Fourteen clinicians were recruited and interviewed from a range of UK NHS hospitals: 12 ICU consultants, 1 professor of respiratory medicine and 1 professor of critical care. All participants had significant experience (>10 years) of diagnosing VAP in UK ICUs.

The interview responses from the participants and the results from the thematic and care pathway analysis are synthesised below, divided into 5 key themes. Supporting quotes for each theme are available in the Supplementary Material, Appendix 3.

### Theme 1: Current practice for the diagnosis of VAP

At the beginning of the interviews, participants were asked to describe the current pathway for diagnosing a suspected VAP in their ICU, including information on general ICU functioning.

#### The intensive care unit (ICU)

ICUs, sometimes called critical care units or intensive therapy units, are specialist hospital wards that care for severely ill patients who are closely monitored, often with a one to one, or one to two, nurse/patient ratio.

Ward rounds occur at least twice daily and involve a multidisciplinary team of physicians, nurses, microbiologists and other allied healthcare providers. During the ward round, the clinical team will review the patient’s clinical characteristics. This review will include the results of the non-specific, routine investigations, which typically include blood, urine and sputum tests.

#### Diagnosing ventilator-associated pneumonia (VAP)

Diagnosing a VAP in the ICU is challenging. The initial suspicion of VAP is based on clinical signs associated with the respiratory system, which are not specific to VAP. The typical clinical signs are high/low temperature, leucocytosis/leukopenia, worsening oxygenation, gas exchange or increasing oxygen requirements, new infiltrates on chest x-ray (CXR) or computerised tomography (CT), suggestive auscultation, general worsening in haemodynamic state, increase in purulent sputum, (colour, thickness and/or frequency), increase in c-reactive protein (CRP), drop in blood pressure, drop in platelet count and others. Besides pneumonia, these signs can also be indicative of atelectasis/collapsed lung/pneumothorax, sepsis, major trauma (e.g. lung or brain injury), cardiogenic pulmonary edema, pulmonary haemorrhage/embolism/fibrosis, cystic fibrosis, pleural effusion, acute respiratory distress syndrome (ARDS), chronic obstructive pulmonary disease (COPD), mucous impaction and other sources of infection. Several clinical scoring systems exist which aim to provide a semi-objective clinical shortcut to the decision to initiate antibiotics for VAP, the Clinical Pulmonary Infection Score (CPIS) being the most widely used and studied [36]. In general, these scores have unsuitable test characteristics compared to microbiological confirmation and the use of CPIS to guide antibiotic decisions is not recommended by the Infectious Diseases Society of America (IDSA) due to pooled sensitivity and specificity of 65% (95% *CI* 61 to 69%) and 64% (95% *CI* 60 to 67%) respectively [37, 38]. Retrospectively applied surveillance definitions are widely used because of the use of VAP as a quality indicator and are useful for benchmarking across populations. They have good face validity, making them tempting reference standards, but they may be gamed through interpretation of radiology [39] or timing of microbiology sampling [40], and exhibit low case concordance [41]. Also, the use of these scoring and recording systems is not always feasible in clinical practice, because it is logistically difficult to reliably record this information in the ICU.

To improve the accuracy of a diagnosis of suspected VAP requires the performance of an invasive diagnostic procedure, where an upper or lower respiratory specimen is collected and sent for microbiological testing. These procedures are sometimes referred to as *special investigations*. The correct special investigation to perform is still a matter of debate, with different ICUs employing different procedures [42]. The main procedures are endotracheal aspirate (ETA), non-bronchoscopic bronchoalveolar lavage (N-BAL), protected specimen brush (PSB) and bronchoalveolar lavage (BAL). The ETA and N-BAL may also be used as non-specific routine investigations.

#### Starting antibiotic treatment for a suspected VAP

The treatment for a suspected VAP is antibiotics. The key driver for starting antibiotics is an overall, holistic deterioration in the clinical signs associated with the respiratory system. No single clinical sign or microbiological result is individually sufficient to initiate the decision to treat. Results from special investigations take time to return (up to 72 h), because of inherent technical limitation in culture-based methods. Consequently, antibiotic treatment is typically started on the basis of clinical signs only. In this situation, treatment is with broad-spectrum antibiotics. The choice of empirical antibiotic is protocolised in an ICU, in accordance with background resistance rates, and influenced by individual patient characteristics. If recent respiratory microbiological isolates are available, they may be used to guide initial treatment, and if microbiology is acquired after initial treatment (i.e. from special investigations), it will be used to tailor a patient’s antibiotics to the causative pathogen, if one is present. The advantages of tailoring antibiotics are several: it leads to more effective killing of bacteria, reduced exposure of patients to unnecessary toxicity (causing *Clostridium difficile*, etc.) [42], reduced risk of developing antimicrobial resistance (AMR) and reduced costs to the unit and healthcare system. There are no disadvantages to appropriately narrowed antibiotics, but if antibiotics are narrowed incorrectly, the bacteria may survive the treatment, with dangerous consequences for the patient. Antibiotics are usually started with a set duration, typically a 5- or 7-day course. This course is strictly completed, unless the antibiotics are narrowed, an alternative diagnosis is confirmed, or if the patient is moving to a palliative mode of care.

#### Stopping antibiotic treatment for a suspected VAP

The key driving factor for stopping antibiotics in a suspected VAP patient is an overall, holistic improvement in the patient, based on the clinical signs associated with the respiratory system. Deciding the exact point at which to stop antibiotics was reported to be extremely challenging, partly because there is no good rule-out or monitoring test for pneumonia, but also because there are no standardised and implemented local NHS guidelines to guide clinical decision-making; see Supplementary Material, Appendix 4, for a review of guidelines for VAP.

Cognitive bias also plays a role in this context. For example, it was reported in the interviews that in the ICU there is a tendency to start with broad-spectrum therapy and maintain this treatment regime, irrespective of whether the patient is getting better or not. If the patient is getting better, then it is assumed that the broad-spectrum antibiotic is working, so they stay broad, and if the patient is getting worse, they may also stay broad and even add on additional antibiotics. It was reported that during handovers between ICU consultants, it is very unlikely that the new consultant will stop antibiotics, even if the plan was to stop them on that day. They are likely to continue for a further 24/48 h to confirm in their own mind that the patient is clinically well enough to stop. It was also reported that antibiotics are often the only treatment available to help an ICU patient recover from illness, so they are check-mated into continuing antibiotics beyond the conventional course length. This is compounded by the ease and cheapness of prescribing antibiotics.

### Theme 2: Current clinical need in VAP diagnostics

The interviews revealed that there is a clinical need for a diagnostic test that provides a more accurate and timely diagnosis of the causative pathogen (or lack of) and for disease monitoring, without the long delays associated with return of results (i.e. 24–72 h for the BAL/PSB), and which is not dangerous to the patient (e.g. the transient reduction in oxygenation, associated with bronchoscopic procedures). When a VAP is present, a diagnostic test with these properties is expected to better facilitate the rationalisation and narrowing of antibiotic prescribing for patients with suspected VAP in the ICU, in comparison to current practice.

### Theme 3: The potential value and role of the OMA platform

#### The potential value of the OMA platform

After discussion of current practice and clinical need, we then presented and discussed the OMA platform and its potential value and role in diagnosing a suspected VAP in the ICU.

It was indicated that the OMA platform’s provision of the real-time gram information, by the bedside, could provide better rationalisation and narrowing of antibiotics—some antibiotics have more potency against Gram-positive than Gram-negative organisms, and vice versa. Stated differently, if the OMA platform can accurately differentiate between Gram-positive and Gram-negative organisms, then the initial empirical, broad-spectrum antibiotics could be narrowed to the particular Gram classification.

Diagnostic tests targeting bacterial infections often give rise to a conceptual question over whether the bacteria detected represents infection or colonisation in the patient [43]; that is, whether the bacteria detected is causing disease or not, respectively—as the lung is non-sterile tissue and bacteria is expected. In addition to revealing the Gram-positive and/or Gram-negative bacteria in the patient lungs, the OMA platform can also identify activated neutrophils, which are associated with inflammation and infection. This information may help the OMA platform differentiate between colonisation and infection.

The OMA platform provides explicit, real-time data through live video-feed to the patient’s bedside. The bacteria and markers of inflammation can be visualised in vivo. It was suggested that this may be more powerful than surrogate markers in influencing decision-making.

#### The potential role of the OMA platform

**Fig. 2:**
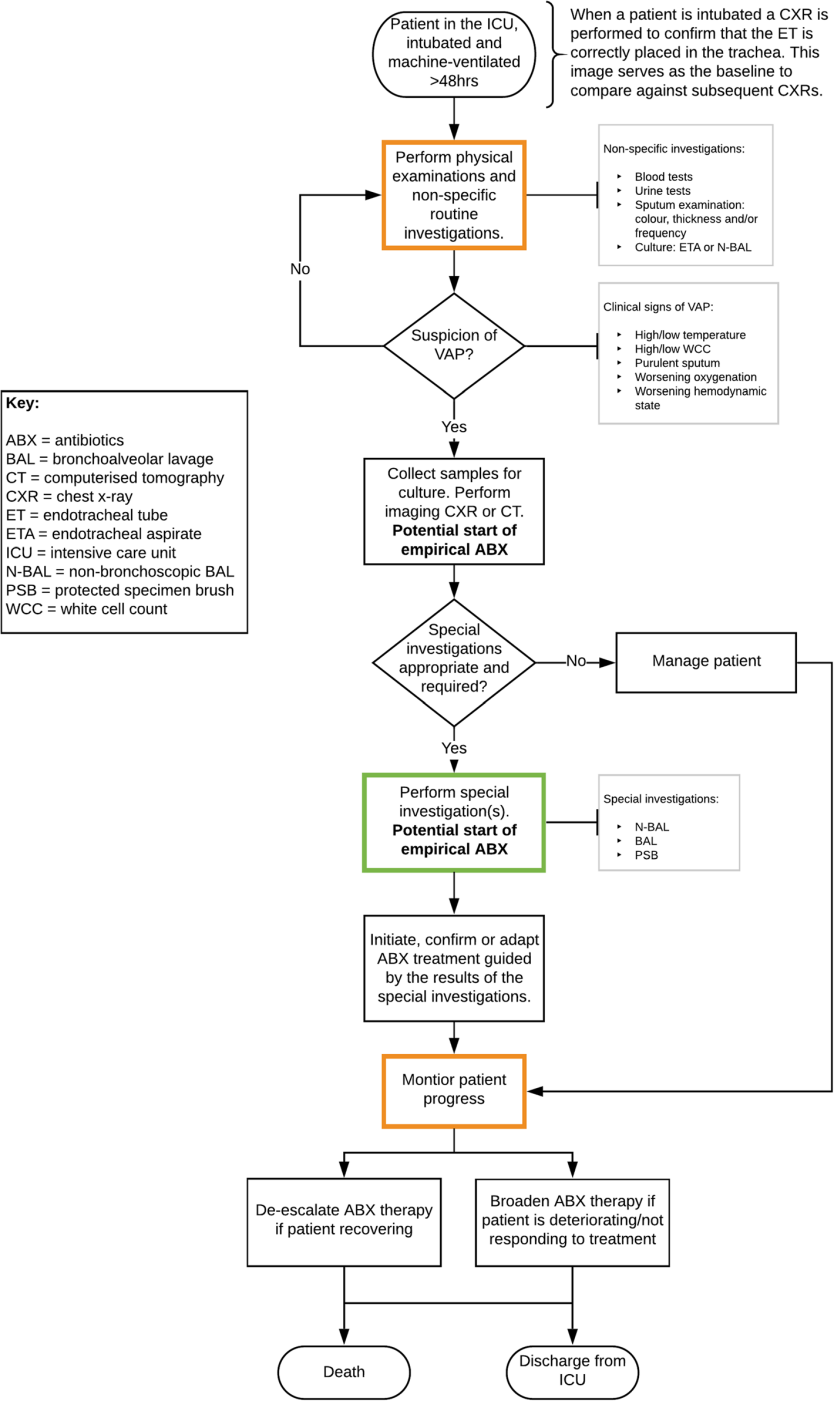
Visualisation of the pathway for diagnosing a suspected VAP in a UK NHS ICU. The coloured boxes in the pathway represent points at which the OMA platform could be used. The green box represents the optimal role for the device, based on the clinical interviews. Here, the OMA platform would be used as a rule-in diagnostic. The orange boxes represent alternative possible roles for the OMA platform, as part of the routine investigations of all eligible ICU patients (top of the pathway) and as a monitoring test in patients with a suspected VAP (bottom of the pathway)

The interviews and the care pathway analysis indicated that the optimal role of the OMA platform would be as a replacement or new/add-on special investigation to diagnose VAP in the ICU. In ICUs that currently use BAL or PSB to diagnose VAP, the OMA platform would be a replacement. In ICUs that do not perform special investigations, the OMA platform would be a new test and an add-on to current practice. In both scenarios, the OMA platform would be used as a rule-in diagnostic, to facilitate rapid and Gram-targeted antibiotic treatment. There may be a secondary role for the OMA platform as a rule-out diagnostic (less likely) and as a surveillance/monitoring device (less likely). These roles, and their advantages and disadvantages, are discussed below and are visually represented in Fig. 2.

##### The OMA platform as a rule-in diagnostic

The OMA platform, as a rule-in diagnostic, would be performed when there is a clinical suspicion of a VAP, when the patient is eligible for a bronchoscopic and transbronchial procedure (i.e. not physically or logistically contraindicated), and (ideally) prior to antibiotic treatment, to maximise the likelihood of visualising the bacteria and neutrophils, and performing a successful culture through the use of the OMA platform’s mini-lavage capabilities.

The real-time, Gram and neutrophil information from the OMA platform may allow the clinician to narrow their antibiotic prescription, from a broad-spectrum, multi-therapy, empirical antibiotic to a Gram-tailored, (ideally) mono-therapy antibiotic. Assuming the OMA platform is sufficiently accurate, this has the potential to kill bacteria more effectively, reduce exposure of patients to unnecessary toxicity, reduce AMR and reduce costs, since more effective treatment should reduce the usage of ICU beds.

##### The OMA platform as a rule-out diagnostic

The OMA platform, as a rule-out diagnostic, would be performed when there is a clinical suspicion of a VAP and when the patient is eligible for a bronchoscopic and transbronchial procedure.

The key advantage to ruling out VAP is that it potentially allows the clinical team to stop or not initiate antibiotics, pushing them to explore alternative diagnoses for the patient. Also, there is a greater clinical need for a rule-out diagnostic, because the pathway to stop antibiotics is more complicated and less standardised across NHS hospitals, than is the pathway to start antibiotics.

The key disadvantage to this role—which is not unique to the OMA platform, but extends to other rule-out tests in NHS ICUs—is that ruling out VAP does not always lead to the stopping or tailoring of antibiotics, because the patient might have an infection elsewhere in the body, including a section of the lung not sampled. As discussed in theme 1, there is a strong bias to continue antibiotics in ICU patients.

##### The OMA platform as a surveillance and/or monitoring device

The OMA platform as a surveillance device would be used as part of the routine investigations. As a monitoring device, it would be used in patients that are being treated for a suspected VAP. In both scenarios, the patient must be eligible for a bronchoscopic and transbronchial procedure.

Using the OMA platform as a surveillance and/or monitoring device could have several advantages, but it was felt that the platform is likely too invasive and expensive to be used repeatedly in ICU patients. The platform, used in these roles, may be appropriate for other non-UK healthcare systems, which perform bronchoscopic procedures more routinely (e.g. parts of Europe, and large teaching hospitals in the USA).

##### Care pathway visualisation

Care pathway visualisation is presented in Fig. 2, below.

### Theme 4: Barriers to adoption

#### Training needs

As with all new diagnostic tests, there will be training needs, but in this case, the need is high because of the complexity and risks of the procedure (discussed in the next section). The training should cover the bronchoscopic procedure (additional trained clinicians may be required if the need for bronchoscopy increases a consequence of the platform introduction), the puncture of the interstitial lung tissue and alveoli (see the next section), the administration of the smart probes, the interpretation of the smart probe data and the collection of the mini-lavage. This would likely require senior respiratory physicians to perform the transbronchial puncture aspect of the procedure, but not all ICU consultants have a respiratory background.

#### Risks of the OMA platform procedure

The transbronchial puncture aspect was highlighted as a potential risk to the patients, because of the risk of causing a pneumothorax. It was stated that the risk of causing a pneumothorax is probably quite low, but the risk level is dependent on the patient’s characteristics. The following patients were identified as possessing a heightened risk of developing a pneumothorax: those patients with ARDS, COPD, sepsis and blood clotting abnormalities and patients requiring high ventilator pressure. It was highlighted that if the OMA platform were to cause a pneumothorax in the early stages of adoption in an ICU, it would likely not be used thereafter. The risk of transbronchial puncture is in addition to the transient interference of oxygenation caused by the bronchoscope and would be increased further if the transbronchial puncture was performed in multiple sites of the lung. The platform’s safety would have to be demonstrated formally and empirically. It is possible that the patients who would get the most out of this platform (e.g. those needing early tailoring of antibiotics) are those that are most at risk of developing complications from the procedure.

#### Increased complexity and effort

The OMA platform, as a bronchoscopic technique, adds an extra layer of complexity to diagnosis in comparison to non-bronchoscopic procedures (e.g. ETAs and N-BALS) and to a clinical diagnosis. The OMA platform requires a concerted effort to perform and interpret its results, and carries inherent risks to patients; therefore, there was some concern in the interviews that clinicians may not use this platform, unless strictly required to by guidelines.

#### Cost

The potential cost-saving element of the OMA platform, associated with more appropriate antibiotic prescribing (discussed in theme 3), is likely to be attenuated by the labour-intensive and highly technical nature of the procedure.

### Theme 5: Evidence requirements to help facilitate successful adoption

To feel confident using the OMA platform, the participants indicated that substantial, high-quality evidence would be required on the diagnostic accuracy, clinical safety and cost-benefits.

It was indicated that the BAL may be a suitable reference standard for evaluating the diagnostic accuracy of the OMA platform. This is consistent with a recent systematic review of VAP diagnostics, which identified BAL culture as the most common reference standard [44]. However, it was noted that clinicians who do not use BALs as their standard of care will be less persuaded to adopt the OMA platform if the study does not compare it with the test that they use (e.g. ETAs or N-BALS). Also, BALs have limitations, in terms of accuracy and safety. Nevertheless, it was still considered to be the optimal choice of comparator for the OMA platform, and since obtaining BAL and using the OMA platform both require bronchoscope and are performed during the same procedure, the recommendation of the study design would be to perform these tests sequentially, one after the other, in patients with suspected VAP, or suspected pulmonary infection, more generally. The OMA platform test would need to be performed first, and BAL second, since the BAL washout would likely interfere with the smart probes, but the reverse would not be the case. Since VAP is a clinical diagnosis, it may also be desirable to construct a composite reference standard [45], but it is essential that the BAL is included in this construct. One possibility here would be to combine a scoring system, such as the CPIS, with a BAL result, and to stratify the diagnosis of the patients into multiple categories (e.g. unlikely, possible, probable VAP). Any reference standard, whether singular or composite, should be a priori selected and/or developed in advance of the diagnostic accuracy study.

Once the accuracy of the platform is adequately demonstrated, a clinical effectiveness/utility trial would be required, where patients would be randomised to the OMA platform or the comparator diagnostic.

Cost-benefit/effectiveness will be required to help facilitate the adoption of the OMA platform in the NHS. The OMA platform and other bronchoscopic procedures cost more than the non-bronchoscopic procedures. Consequently, it will need to be demonstrated that the OMA platform provides value for money and is affordable, which could stem from better, more tailored patient care and reducing ICU time.

## Discussion

In this study, we performed qualitative, semi-structured interviews with clinical experts. We used the information in these interviews to perform a care pathway analysis of current practice for diagnosing a suspected VAP in the UK NHS ICUs. We also explored the potential value and role of the OMA platform.

The care pathway analysis revealed that making a diagnosis of VAP is extremely difficult, primarily because the clinical signs associated with VAP overlap with several other diseases. To improve the accuracy and certainty of a VAP diagnosis, a special investigation may be performed (e.g. ETA, N-BAL, BAL, PSB), where, depending on the method, an upper or lower respiratory specimen is collected and sent for culture. These investigations vary in accuracy, invasiveness and cost. Time delays in receiving these results mean that the decision to treat (predominantly, with antibiotics) is based on clinical signs only. Consequently, antibiotics are typically empirical and broad spectrum.

The key driver for starting antibiotics is an overall, holistic deterioration in the clinical signs associated with the respiratory system. Antibiotics will be narrowed where possible, when microbiology becomes available. Antibiotics are usually started with a set duration, typically a 5- or 7-day course, and this course is typically completed.

The key driving factor for stopping antibiotics is an overall, holistic improvement in the patient, based on the clinical signs associated with the respiratory system, including microbiological information. Deciding the exact point at which to stop antibiotics was reported to be extremely challenging, partly because there is no good rule-out test for pneumonia and no standardised guidelines. It was also indicated that cognitive biases might affect the decision to stop antibiotics. Biases have previously been demonstrated to be present in clinical decision-making [46, 47] and antibiotic prescribing behaviour [48].

The interviews revealed that there is a clinical need for a diagnostic test that provides a more accurate and timely diagnosis of VAP, which is not dangerous to the patient, and which has properties that better facilitate the rationalisation and narrowing of antibiotic prescribing for patients.

Based on the care pathway analysis, it was determined the OMA platform could potentially satisfy important aspects of the above clinical need. The OMA platform’s provision of real-time gram information, by the bedside, could provide better rationalisation and narrowing of antibiotics, which may be more powerful than currently used surrogate markers in influencing clinical decision-making. Also, the platform’s ability to identify activated neutrophils may also help differentiate between bacterial colonisation and infection. Although the focus of this study was VAP, these value propositions for the OMA platform may be equally applicable to all forms of bacterial pneumonia experienced in the ICU.

### Limitations

A larger sample size of interviewees may have been advantageous in eliciting different views on the VAP diagnostic pathway and the OMA platform, but the decision was made to stop at 14 because data saturation was reached. Consequently, it was felt that further consumption of clinician time would have been gratuitous.

No formal, quantitative assessment of intercoder reliability was performed in this study. The coders, WSJ and JS, are experienced in qualitative diagnostic pathway analysis and have worked together previously to form consensus on themes. They independently coded the pilot interviews, compared results and determined that they were in alignment. This alignment was, to a large extent, facilitated by the form of the interview schedule, which was a priori divided down thematic lines, involving fairly closed and specific questions. Finally, WSJ, JS, and the rest of the study team were in constant conversation on the study interview answers. However, there is likely some value to performing some form of quantitative assessment of reliability [49], and subsequent work will consider and innovate ways in which this could be performed in a meaningful fashion for this narrow type of qualitative analysis.

Care pathways are not static. When new practice and technology become available, as budgets and healthcare systems change, so too do pathways. Therefore, the pathway explicated in this article will only hold for a certain period of time. This is a problem for all forms of care pathway analysis and work that builds on these (e.g. health economics, diagnostic accuracy studies, …).

## Conclusions

Diagnosing a VAP in the ICU is challenging. The initial suspicion of VAP is based on clinical signs associated with the respiratory system (including bloods and CXRs), which are not specific to VAP and do not allow for the tailored antibiotic treatment. To improve the accuracy, or to reduce the uncertainty, requires an invasive diagnostic procedure, where an upper or lower respiratory specimen is collected and sent for microbiological testing. These procedures vary in safety, accuracy and influence on clinical decision-making.

There is a clinical need for a diagnostic test that provides a more accurate and timely diagnosis of the causative pathogen (or lack of) and for disease monitoring. When a VAP is present, a diagnostic test with these properties would better facilitate the rationalisation and narrowing of antibiotic prescribing in comparison to current practice. The care pathway analysis revealed that the OMA platform would address this aspect of the clinical need, but further evidence would be required on its accuracy, safety and cost-benefit.

### Future recommendations

Further research into the cognitive biases involved in antibiotic decision-making in the ICU would be informative for clinicians and developers.

## Supplementary Information

**Additional file 1.** Appendices.

## Data Availability

Key quotes from the interviews are provided in the Supplementary Material Appendices. No additional data are available.

## References

[1] The National Institute for Health and Care Excellence (NICE), *Pneumonia in adults: diagnosis and management.* 2019. 31940163

[2] Anand N, Kollef MH (2009). The alphabet soup of pneumonia: CAP, HAP, HCAP, NHAP, and VAP. Semin Respir Crit Care Med.

[3] Koulenti D, Tsigou E, Rello J (2017). Nosocomial pneumonia in 27 ICUs in Europe: perspectives from the EU-VAP/CAP study. Eur J Clin Microbiol Infect Dis.

[4] Niederman MS (2010). Hospital-acquired pneumonia, health care-associated pneumonia, ventilator-associated pneumonia, and ventilator-associated tracheobronchitis: definitions and challenges in trial design. Clin Infect Dis.

[5] Ferrer M, Torres A (2018). Epidemiology of ICU-acquired pneumonia. Curr Opin Crit Care.

[6] Spencer RC (1994). Epidemiology of infection in ICUs. Intensive Care Med.

[7] Vallés J, Pobo A, García-Esquirol O, Mariscal D, Real J, Fernández R (2007). Excess ICU mortality attributable to ventilator-associated pneumonia: the role of early vs late onset. Intensive Care Med.

[8] Heyland DK (1999). The attributable morbidity and mortality of ventilator-associated pneumonia in the critically ill patient. The Canadian Critical Trials Group. Am J Respir Crit Care Med.

[9] Safdar N, Dezfulian C, Collard HR, Saint S (2005). Clinical and economic consequences of ventilator-associated pneumonia: a systematic review. Crit Care Med.

[10] Kollef MH, Hamilton CW, Ernst FR (2012). Economic impact of ventilator-associated pneumonia in a large matched cohort. Infect Control Hosp Epidemiol.

[11] Rello J, Ollendorf DA, Oster G, Vera-Llonch M, Bellm L, Redman R, Kollef MH, VAP Outcomes Scientific Advisory Group (2002). Epidemiology and outcomes of ventilator-associated pneumonia in a large US database. Chest.

[12] Balogun B. Coronavirus: ventilator availability in the UK, H.o.C. Library; 2020. https://commonslibrary.parliament.uk/research-briefings/cbp-8904/.

[13] Mahase E (2020). Covid-19: most patients require mechanical ventilation in first 24 hours of critical care. BMJ.

[14] Akram AR (2018). In situ identification of Gram-negative bacteria in human lungs using a topical fluorescent peptide targeting lipid A. Sci Transl Med.

[15] Chan YR, Morris A (2007). Molecular diagnostic methods in pneumonia. Curr Opin Infect Dis.

[16] Drabińska N, de Lacy Costello B, Hewett K, Smart A, Ratcliffe N (2019). From fast identification to resistance testing: volatile compound profiling as a novel diagnostic tool for detection of antibiotic susceptibility. TrAC Trends Anal Chem.

[17] Hellyer T, Simpson J (2014). Biomarker-based exclusion of ventilator-associated pneumonia: a multicentre validation study. Crit Care.

[18] Hellyer TP, Conway Morris A, McAuley DF, Walsh TS, Anderson NH, Singh S, Dark P, Roy AI, Baudouin SV, Wright SE, Perkins GD, Kefala K, Jeffels M, McMullan R, O'Kane CM, Spencer C, Laha S, Robin N, Gossain S, Gould K, Ruchaud-Sparagano MH, Scott J, Browne EM, MacFarlane JG, Wiscombe S, Widdrington JD, Dimmick I, Laurenson IF, Nauwelaers F, Simpson AJ (2015). Diagnostic accuracy of pulmonary host inflammatory mediators in the exclusion of ventilator-acquired pneumonia. Thorax.

[19] Jung JH, Lee JE. Real-time bacterial microcolony counting using on-chip microscopy. Sci Rep. 2016;6(1):–21473. 10.1038/srep21473. 10.1038/srep21473 PMC4763285 26902822

[20] Mills B, Bradley M, Dhaliwal K (2016). Optical imaging of bacterial infections. Clin Transl Imaging.

[21] Morris AC (2018). Management of pneumonia in intensive care. J Emerg Crit Care Med.

[22] Slupsky CM (2011). Nuclear magnetic resonance-based analysis of urine for the rapid etiological diagnosis of pneumonia. Exp Opin Med Diagn.

[23] The Proteus Interdisciplinary Research Collaboration is funded by the Engineering and Physical Sciences Research Council (EPSRC). 2017; Available from: https://proteus.ac.uk/.

[24] Korevaar DA, Gopalakrishna G, Cohen JF, Bossuyt PM (2019). Targeted test evaluation: a framework for designing diagnostic accuracy studies with clear study hypotheses. Diagn Prognostic Res.

[25] Charman S, Okwose N, Maniatopoulos G, Graziadio S, Metzler T, Banks H, Vale L, MacGowan GA, Seferović PM, Fuat A, Deaton C, Mant J, Hobbs RFD, Jakovljevic DG (2019). Opportunities and challenges of a novel cardiac output response to stress (CORS) test to enhance diagnosis of heart failure in primary care: qualitative study. BMJ Open.

[26] De Bleser L (2006). Defining pathways. J Nurs Manag.

[27] Graziadio S (2020). How to ease the pain of taking a diagnostic point of care test to the market: a framework for evidence development. Micromachines (Basel).

[28] Kinsman L (2010). What is a clinical pathway? Development of a definition to inform the debate. BMC Med.

[29] Panella M, Vanhaecht K (2010). Is there still need for confusion about pathways?. Int J Care Pathways.

[30] Saunders B, Sim J, Kingstone T, Baker S, Waterfield J, Bartlam B, Burroughs H, Jinks C (2018). Saturation in qualitative research: exploring its conceptualization and operationalization. Qual Quant.

[31] Braun V, Clarke V (2006). Using thematic analysis in psychology. Qual Res Psychol.

[32] Castleberry A, Nolen A (2018). Thematic analysis of qualitative research data: is it as easy as it sounds?. Curr Pharm Teach Learn.

[33] Maguire M, Delahunt B. Doing a thematic analysis: s practical, step-by-step guide for learning and teaching scholars; 2017. https://ojs.aishe.org/index.php/aishe-j/article/view/335.

[34] Nowell LS, Norris JM, White DE, Moules NJ. Thematic analysis: striving to meet the trustworthiness criteria. Int J Qual Methods. 2017;16(1). 10.1177/1609406917733847.

[35] Gale NK, Heath G, Cameron E, Rashid S, Redwood S (2013). Using the framework method for the analysis of qualitative data in multi-disciplinary health research. BMC Med Res Methodol.

[36] Pugin J, Auckenthaler R, Mili N, Janssens JP, Lew PD, Suter PM (1991). Diagnosis of ventilator-associated pneumonia by bacteriologic analysis of bronchoscopic and nonbronchoscopic “blind” bronchoalveolar lavage fluid. Am Rev Respir Dis.

[37] Kalil AC, Metersky ML, Klompas M, Muscedere J, Sweeney DA, Palmer LB, Napolitano LM, O'Grady NP, Bartlett JG, Carratalà J, el Solh AA, Ewig S, Fey PD, File TM, Restrepo MI, Roberts JA, Waterer GW, Cruse P, Knight SL, Brozek JL (2016). Management of adults with hospital-acquired and ventilator-associated pneumonia: 2016 Clinical Practice Guidelines by the Infectious Diseases Society of America and the American Thoracic Society. Clin Infect Dis.

[38] Shan J, Chen HL, Zhu JH (2011). Diagnostic accuracy of clinical pulmonary infection score for ventilator-associated pneumonia: a meta-analysis. Respir Care.

[39] Walsh TS, Morris AC, Simpson AJ, Ventilator V (2013). associated pneumonia: can we ensure that a quality indicator does not become a game of chance?. BJA Br J Anaesth.

[40] Craven TH, Wojcik G, McCoubrey J, Brooks O, Grant E, Keating S, Reilly J, Laurenson IF, Kefala K, Walsh TS (2020). Ventilator-associated pneumonia surveillance using two methods. J Hosp Infect.

[41] Craven TH, Wojcik G, McCoubrey J, Brooks O, Grant E, Reilly J, Laurenson IF, Kefala K, Walsh TS (2018). Lack of concordance between ECDC and CDC systems for surveillance of ventilator associated pneumonia. Intensive Care Med.

[42] Arulkumaran N, Routledge M, Schlebusch S, Lipman J, Conway Morris A (2020). Antimicrobial-associated harm in critical care: a narrative review. Intensive Care Med.

[43] Dani A (2014). Colonization and infection. Cent Eur J Urol.

[44] Al-Omari B (2021). Systematic review of studies investigating ventilator associated pneumonia diagnostics in intensive care. BMC Pulm Med.

[45] Naaktgeboren CA, Bertens LCM, Smeden M, Groot JAH, Moons KGM, Reitsma JB (2013). Value of composite reference standards in diagnostic research. Bmj.

[46] O'Sullivan ED, Schofield SJ (2018). Cognitive bias in clinical medicine. J R Coll Phys Edinb.

[47] Saposnik G, Redelmeier D, Ruff CC, Tobler PN (2016). Cognitive biases associated with medical decisions: a systematic review. BMC Med Inform Dec Making.

[48] Warreman EB, Lambregts MMC, Wouters RHP, Visser LG, Staats H, van Dijk E, de Boer MGJ (2019). Determinants of in-hospital antibiotic prescription behaviour: a systematic review and formation of a comprehensive framework. Clin Microbiol Infect.

[49] O’Connor C, Joffe H (2020). Intercoder reliability in qualitative research: debates and practical guidelines. Int J Qual Methods.

