# Supplementary material for: Diagnosing ventilator-associated pneumonia (VAP) in UK NHS ICUs: the perceived value and role of a novel optical technology
Source: Diagn Progn Res. 2022 Feb 10;6:5. doi: 10.1186/s41512-022-00117-x (PMC8830125; doi:10.1186/s41512-022-00117-x)
Supplement: Supplementary file 1 — Additional file 1. Appendices. [file 41512_2022_117_MOESM1_ESM.pdf]

Appendices

Contents

Appendix 1 – Interview topic guide 2
Appendix 2 – Framework analysis approach to qualitative data 3
Stage 1: Transcription of audio recording 3
Stage 2: Familiarisation with the content of the interviews 3
Stage 3: Coding 3
Stage 4: Developing a working analytical framework 3
Stage 5: Applying the analytical framework 3
Stage 6: Charting the data into the framework matrix 3
Stage 7: Interpreting the data 3
Appendix 3 – Supporting quotes for thematic and care pathway analysis 4
Theme 1: Current practice for the diagnosis of VAP 4
Theme 2: Current clinical need in VAP diagnostics 18
Theme 3: The potential value and role of the OMA platform 19
Theme 4: Barriers to adoption 21
Theme 5: Evidence requirements 24
Appendix 4 – Guideline review 26
Appendix 5 – COREQ Checklist 30
References 31

Appendix 1 – Interview topic guide

1. **Where do you work and your job role?**
    a. Can you give us a brief overview with your experience with VAP?
    b. How big is the hospital (Beds in ICU, size of hospital)?

Now I would ask you to take a look at the PDF with the VAP pathway. Do you have this in front of you?

This is a draft visualisation of the pathway that we think a suspected VAP patient follows in the ICU. As I said, it is only in draft form, developed on the basis of conversations with clinicians that we collaborate with, and a small pilot study.

What we would like you to do is work your way through this pathway, from top to bottom, box by box, to see if it is representative of your current understanding and practice. Also, if you could talk us throughout laud your thinking at each stage that would be very useful for the recording.

After we have established current practice, then we introduce the product with a video.

2. **Do you think this technology would alter your clinical decision-making?**
    a. Would this technology help you for monitoring purposes (vs diagnostic)?

3. **What do you perceive to be the advantages of this technology?**
    a. So, where do you think it might fit in the pathway, assuming that it still requires a bronchoscope at the moment? Do you think there is anywhere in particular that you might think it might be useful?
    b. What expertise do you think is required for the procedure?

4. **What would be your main concerns with this technology?**
    a. Are there solutions to these barriers?
    b. Transbronchial puncture, if the technology had an associated risk similar to BAL, would you feel comfortable in using it?
    c. Another thing to mention on this front is that they are going to be testing multiple sites within the lung. In your opinion that's a good thing or a bad thing or how many sites perhaps they should be looking at?

5. **What type of evidence would you want to see to feel confident in using this technique, and…?**
    a. Accuracy level (What would be the minimal level of Se and Sp for you to feel comfortable using the technology?) and cost effectiveness (What would be a feasible cost for this test?).
    b. They're planning a clinical trial at the moment, and the information from interviews will help them with the design. So, we're trying to work out what we're going to compare it to, as a reference/gold standard. So, did you have any idea on that? What do you suggest that the new testing procedure should be compared against? We are considering developing a composite reference standard…

Appendix 2 – Framework analysis approach to qualitative data

Gale et al. outline 5 stages to framework analysis (p. 4-5):

Stage 1: Transcription of audio recording

We paid a professional service, UK Transcription [1], to construct verbatim transcriptions of the interview audios. Prior to using this service, we established the requisite non-disclosure agreements with UK Transcription, to ensure that the data from the interview audios was securely handled.

Stage 2: Familiarisation with the content of the interviews

Once the interviews were transcribed, WSJ and JS spent several hours familiarising themselves with the interview content, by examining and making notes on the interview transcripts, whilst listening and re-listening to the audio recordings, which helps to get a richer understanding of meaning.

Stage 3: Coding

After WSJ and JS had familiarised themselves with the interview content, they carefully reread the transcripts, line by line, applying (or indexing) predetermined codes to important sentences and paragraphs. These codes where *a priori* selected as important to the study aims. The process of coding allows data to be systematically organised and compared. WSJ and JS independently coded the first two pilot transcripts, to synchronise their coding approach. The coding of transcripts was performed digitally, using computer-assisted qualitative data analysis (CAQDAS). We used R-based Qualitative Data Analysis (RQDA) [2], which is a free package in R programming language [3].

Stage 4: Developing a working analytical framework

After coding 5 transcripts, WSJ and JS reviewed the efficacy of the pre-determined codes and made alterations as required. Codes are grouped together under specific themes.

Stage 5: Applying the analytical framework

The analytical framework, of codes and overarching themes/categories is then applied to organise the data so they are ready accessible and ready for analysis.

Stage 6: Charting the data into the framework matrix

Each interview generated approximately 15 pages of transcription. We used RQDA to organise and summarise this large quantity of data down to manageable size.

Stage 7: Interpreting the data

Once the transcripts were coded and organised into themes, we then began the interpretation of the data, whilst simultaneously constructing a narrative synthesis.

Appendix 3 – Supporting quotes for thematic and care pathway analysis

Theme 1: Current practice for the diagnosis of VAP

'…so the patients in the ICU… ventilated for more than 48 hours would be a conventional starting point for consideration of VAP.'

'Obviously, people can get pneumonia before 48 hours. Whether you define it- it is completely arbitrary saying it's 48 hours, but that is a recognised threshold point for unit acquired infection… That is our local definition. Most VAPs, though, are endogenously acquired from the microbes in that patient, and they will be there at 12 hours and at 60 hours. It is arbitrary, but you've got to have some kind of definition for research purposes and that is ours; 48 hours is our definition.'

'[VAP]…patients mechanically ventilated for more than the 48 hours. I think this is largely used to distinguish it between other types of pneumonia, most commonly hospital-acquired pneumonia.'

'The patient is on ICU… and intubated, so that's going to conform for ventilator-associated pneumonia… for more than 48 hours. Again, that's in the definition, so that's all fine.'

'Most patients who would come, who are intubated, after intubation will have a chest X-ray immediately. Then, obviously, with the definition of a ventilator associated pneumonia, more than 48 hours, you're looking at new changes; you're looking for new changes on the imaging.'

'Mechanically ventilated for over 48 hours, that's a reasonable group of patients to target. There's a comment about them most likely having a baseline chest x-ray, that's quite likely as well in my experience.'

'…patient being in the ICU, intubated and ventilated more than 48 hours, which seems entirely sensible because less than that and you would be suspicious that it was a community-acquired infection that had simply manifested itself [slightly later]… [a baseline] chest x-ray, which seems entirely reasonable. I think pretty much everybody would have that after intubation. It's not unreasonable to count it as a baseline but it's not the same thing as saying it's normal. It might be quite abnormal.'

'You've got a starting point of patients in the ICU; intubated and mechanically ventilated. I see this note about a chest x-ray is performed. My usual practice, and the unit's practice where I work, would be to perform a chest x-ray post-intubation in a patient again, as you say, to confirm the position of the tube in the trachea and that does also act as a baseline. We would always perform a post-intubation chest x-ray in our patients as described there.'

'So, they have to be ventilated for greater than 48 hours, otherwise we assume it's community, or non-ventilator-associated pneumonia. So, yes. It's patients with intubations currently ventilated for greater than 48 hours. And yes, we always perform a chest X-ray, once the patient is intubated.'

'Yes. Absolutely, yes. Absolutely, 100%. We have a baseline chest x-ray done when somebody is intubated, absolutely. Sometimes, obviously, they're intubated in the emergency department, but we will have a baseline chest x-ray, definitely.'

'Yes. It's hard to say 'every', but every patient who has been intubated recently should have a chest X-ray as a baseline investigation... They will have at least had their first chest X-ray. Probably chest X-rays before that but definitely one after they had been intubated… I guess there are some patients that might have had CT rather than chest X-ray, if for some reason they had come through say unconscious and then scanned for other reasons. You might not do a chest X-ray. So I guess there are occasionally patients who might have had CT of the chest rather than a chest X-ray.'

'A chest x-ray is usually performed at baseline, although we are increasingly using ultrasound in terms of diagnostics, moving forwards with the new technology, I think there should be a consideration of ultrasound diagnostics as well as more traditional chest x-ray imaging. [It gives results then], ultrasound is non- there's no radiation and it's immediately available to give you a result and it can do all the things that chest x-rays can do… I just suspect that, going forwards, as ultrasound technology becomes miniaturised and the fidelity of the ultrasound gets higher, that over time ultrasound will increase and chest x-rays will decrease. But having said that, chest x-rays are more immediately available because we have digital x-ray machines, so we can take the x-ray and literally look at it one second later. So there's still a place for that.'

'So there's an initial chest X-ray, there is an initial set of screening bloods for all admissions, and blood cultures as well. And the blood tests are done daily. The blood cultures or sputum cultures, or any other cultures, are done as required, and it's generally based on the clinical condition of the patient.

'somebody who has had a clear chest x-ray when they came in, they've been in for two or three days on a ventilator for another reason, and they start to get new infiltrates on their chest x-ray or start to become pyrexial, or their white cell count goes up, yes, we would have a suspicion.'

'…we do a ward round twice a day. The ward round in the morning is specifically, the patient will be examined to see if they have changed in any way…'

'I don't tend to use a scoring system. I tend to pull all the data in and have a think about it. Generally, because we do two ward rounds every day, there's always a lot of discussion about how every patient's doing. There's a lot of synthesis of information if you like, coming in from all different parameters that we would be thinking about.'

'The prescribing behaviour would be, it's very driven. We obviously have daily microbiology rounds. We've got a good relationship with the

microbiologists and a clear protocol for how to treat. If it's a medical patient coming in with ventilatory failure and an early pneumonia, then we'd go down our standard community acquired pneumonia antibiotics. If it develops on the unit, then we would usually start – if we've got an isolate from a previous m-BAL, we would target that organism. If they've grown staph or haemophilus of a gram negative, we'd tailor it accordingly. If we didn't have any isolates and there was a VAP, then we'd probably start relatively broad with something like taz or mero. Probably taz because our microbiologists like us to avoid carbapenems if we can. If we've got some other organisms, we might be happy saying, "Well at the moment co-amoxiclav is sufficient based on the organisms we've isolated." We might give a shot of intravenous gentamycin if there were concern about resistant gram-negative organisms initially. It would really depend on the timing, previous antibiotic exposure and if we'd grown something in the non-directed BAL.'

'So, all patients would get blood work done every day. If they had pyrexia they would get blood cultures done.'

'We have a set of routine investigations that are in all our patients on ventilators. Our level three patients will all have routine investigations every day. Non-specific investigations. And we routinely do CRP. We should have CRP every day. Along with a white cell count. I guess we've got two things that are done routinely. We also do on our unit routine surveillance non-directed bronchial lavage on our intubated patients. We do that on a Monday, Wednesday and Friday. They all have routine sampling. That's a special investigation, but it's done regardless of your clinical state if you're intubated… Their cultures are done as a routine, although obviously we will do them targeted if we think the patient has deteriorated. Chest X-rays are not done routinely but would be done if a patient's clinical condition has deteriorated.'

'…looking at… a full blood count, raised white cell counts and presence of other perhaps positive inflammatory markers, such as CRP, or other markers, for example, that some centres we don't use regularly – things such as procalcitonin – for identification of bacterial infection… culture from aspirates from an endotracheal tube.'

'So, CRP and white cell count… would be part of a standard daily audit. So, yes, they would be done daily, no matter what. With regards to the cultures, the sputum cultures are done weekly as standard. Obviously, on top of that if there are any changes in the patient's condition… you might do more sputum cultures.'

'…history, physical examination and investigations and any special investigations that might be done. Again yes, that's right; one would hope that's something that would come with patients from the ward or from A&E and then obviously regularly repeated again as necessary in intensive care... They would be done on an as-needed basis. We wouldn't routinely do say, blood cultures on a patient, but if we were suspicious of infection for whatever reason, then obviously blood cultures would be an integral part of the investigation.'

'[patient history] usually done in the ward but also reassessed in ICU which is history, physical examination, special investigations, yes. We would, if we were admitting a patient and mechanically ventilating them then depending on the setting, so if we were taking a medical patient primarily for ventilatory failure and intubating them, we would try to do package. If they've got a pulmonary infiltrate, we do a package of – at the current season we do flu swaps, we would do some urinary antigens for pneumococcus and legionella and we would usually do an HIV test as well and some blood cultures looking for the aetiology of ventilatory failure with a pulmonary infiltrate. That might be a bit different if the patient was immunosuppressed, but that's sort of our package.'

'The one thing that we do, do that I see is not really on this pathway; we do, do surveillance non-directed bronchoalveolar lavage. We do that on Mondays and Thursdays… basically, in any intubated patient, ET tube or tracheostomy. Unless there's a contraindication. Unless they've got ARDS and a high level of PEEP and you don't want to do that, or that you feel they're too unstable.'

'Yes. Definitely anything to do with bloods done, blood cultures, we'd only do if somebody was pyrexial and imaging for the chest x-ray, we would get that if there had been a change in somebody's clinical condition. So, somebody has been stable but they look like they're getting worse and we want to know why they're getting worse. So, they would get a chest x-ray at that point.'

'We wouldn't necessarily do daily chest x-rays if somebody was stable… but if their oxygen level is getting worse, or the amount of support they are requiring is going up, then we'd… want to see why they're becoming a lot more breathless.'

'So, there are a number of things you're looking at. So, firstly, you're looking at deterioration in their respiratory function in the first place. Have they started to produce more secretions? Are their lungs stiffer? Are their blood gases worse? Then you would look at indicators of inflammation, such as, is the white cell count higher? Has their CRP risen, despite treatment? I guess then you would look to investigate the other investigations. So, has the chest X-ray changed since admission significantly? So, I guess there are three criteria; respiratory function, radiological findings, and evidence from blood tests of worsening or primary infection, or inflammation. But it's very difficult. The diagnosis of nosocomial pneumonia is very subjective. Yes, it's very subjective, in my view.'

''Blood cultures tend to be done if the patient is deteriorating and/or has a pyrexia that's over 38.3.

'The first thing I would see really is, is there evidence of clinical deterioration with the respiratory system, with the lungs of the primary cause. So, I'm looking for signs.'

'Well, microbiological testing is notoriously poor at both diagnosing VAP, about separating what's just colonisation from infection. Even if there is infection, it's notoriously difficult about (a) growing anything, and (b) getting anything useful about it. So, most of the time, it's clinical suspicion, I would say. I think we get confirmation by microbiological testing less than half the time.'

'We would suspect VAP in any patient who develops a febrile episode in whom the daily bloods show an increase in white count or CRP. Nurses reporting change in sputum purulence or lots of secretions. Increase in oxygen requirement. Clinical signs of new crackles in the chest. Of course, we do a chest x-ray and look for any evidence

of radiological change compared to the old one. Fairly classical things. I suppose we'd ask it in any patient. Each day we'd let the thought of whether they'd developed a VAP go through our mind.'

'Well, probably the first thing would be the nurses saying the patient's oxygen requirements have increased. Because they'll spot that immediately and they'll tend to bring that to our attention. Or, it might be a temperature, it might be a fever. Or, it might be that we spot on the routine full blood count, a rise in the white cell count. Now, bearing in mind that patients have all sorts of different pathologies, none of those would be specific for ventilator associated pneumonia, but they would raise the suspicion that that would be in the differential diagnosis... Well, I mean all sorts of possibilities. It could be an aspiration pneumonia. It could be pulmonary oedema. It could be ARDS. There's quite a lot of different things basically. VAP would be one of them, obviously.'

'…patients in intensive care, it's one-to-one nursing. So, it's one nurse for one patient. So, the nurses are constantly evaluating them, and every hour the patients get observations recorded, and every hour they get a temperature, for example. They'll have their blood pressure and their heart rate, and everything, recorded. Maybe a few hours, they take a sample of blood from an artery. You've got a little device in your artery, usually, when you're sick, called an arterial line. You can take blood from that, and they run that through an analyser and you see exactly what the oxygen level is on your blood and they will guide the- they will titrate, up or down, the oxygen dependent upon that. So, that happens several times a day. So, a combination of your temperature and your heart rate, your blood pressure and your oxygen levels. So, the nurses titrate things up or down, yes, as they go along.'

'…a new temperature which might indicate a new infection.'

'…somebody who has got a pyrexia, somebody whose white cell count is going up, somebody whose oxygen requirements are going up, who has been stable, and if you do a chest x-ray, somebody who has got [an infiltrate] in their chest x-ray. We use helix criteria, which you're probably familiar with.'

'If you've got a temperature, if you've got a very high fever, white count, sputum, worsening oxygenation, worsening haemodynamic status, infiltration, you're probably going to call that a VAP in whatever patient.'

'…a high or low temperature as the first indicator, and the worsening oxygenation. So, there could be a source of infection elsewhere, as well as within the lungs. You can target that down a little bit more with positive sputum culture, or identification of possibly infected looking sputum, purulent sputum, and obviously, new changes on a chest X-ray.'

'Absolutely. So, high and low temperature, absolutely. High or low white cell count, absolutely. Usually, you would be more in common to see a high temperature and a high white cell count rather than a low white cell count or a low temperature but, yes, I agree with both of them… sputum, absolutely. Worsening oxygenation, totally agree. Worsening haemodynamic status, possibly if they were becoming very sick but it wouldn't necessarily be a first feature. Infiltrates on a chest x-ray, yes…'

'To be honest, my own practice is I would look to see, it would be a clinical judgement, really. So, for example, if the patient had had just one spike of temperature and their chest x-ray had only changed minimally, you weren't getting much in the way of sputum up, I might hang fire a little bit, just to see if it was a bit of a blip. If somebody was persistently pyrexial or if somebody had had a rapid rise in their inflammatory markers. So, for example, their CRP or a white cell count, or if they were becoming haemodynamically unstable, I would hit them with antibiotics quickly. So, it's really a clinical judgement, I would say.'

'Context. So in a brain injured patient, for example, they're all going to get a VAP, as opposed to somebody who is perioperative. If you've got a temperature, if you've got a very high fever, white count, sputum, worsening oxygenation, worsening haemodynamic status, infiltration, you're probably going to call that a VAP in whatever patient. But if it's in a patient who is immunosuppressed or in a group of patients that are likely to get VAP, this could prolong ventilation etc. Then you're more likely to call it, probably. So context is important.'

'The answer is that confirming is very difficult. The best way to is to have an educated guess. Even at the best of times, it's going to be a strong possibility but a certainty is quite difficult because sputum cultures can be false positives. There's often [something in the] airway that may contaminate the samples. Then, if it's negative, that doesn't mean there's nothing there. The more… signs that you've got, the more confident you are that you are likely to have a ventilator-associated pneumonia. You can never be absolutely sure. With regards to further microbiology at the moment. So most of the time, yes a [protected] sputum culture from endotracheal aspirates, in our hospital.'

'Similarly, with the white cell count, the purulent sputum is a more specific positive indicator potentially of a respiratory infection, but clearly, it's not directly from, for a better description, deep in the lungs, that's been aspirated; and then worsening oxygenation, yes. Clearly, that is suggesting if oxygen requirements are going up that there's something going on with the lungs. Again, it suggests something's going wrong. Again, it's not specific. Worsening haemodynamic state; more likely to reflect a general systemic infection, a sepsis, rather than specifically a VAP.'

'It will be a deterioration in the patient. That's normally a worsening of their blood gases. Normally their oxidation. Worsening oxidation I think is probably your main trigger… you then start to take in the other bits of data. So then you would say, "What's the sputum load like? Has the sputum changed in its nature?" Colouration. Thickness. Those bits come into it then. Then I guess you would look at the routine investigations. The white cell count. The CRP trend. Then you may bring in a chest X-ray… Probably clinically the first things, I guess, would be oxygenation. Oh, I see you've got temperature on that. Again, that might be something. If the patient is spiking a fever, the white cells are up, the sputum is more productive, then these are all suspicions that something is going on in their chest.'

'[What are the clinical features in an ICU patient that first make you suspect a VAP?] I think the two biggest ones would be the increasing [appearance] of sputum and deterioration in gas exchange – so worsening oxygenation, or requirement for increasing oxygen

concentration in order to maintain that oxygenation'

'We do use other markers of infection. White count is not particularly good, C-reactive protein is a little bit better, platelet count is quite sensitive. So if your platelet count suddenly drops, that normally is an indication of acute infection and if there's a feature suggesting that the source affected is the chest, then that would make you think of VAP.'

'…blood pressure going down could be indicative of a problem anywhere in the body. There could be another source, but pneumonia has caused sepsis. So, that's why I think it's important to include it.'

'…we have tried to use is the CPIS scoring system but, again, I think we've… You know, I'll be honest with you, we've put that on our daily review charts for the patients. Again, to get that to be filled in reliably, consistently, every day, is mighty difficult.'

'[Do you use a scoring system to capture these or record these?]… I think on a clinical basis, it's more in your mind. On a data basis, all patients are scored for VAP in Scottish ICUs using Helix criteria.'

'We don't officially have one as part of our protocol, but when we're doing research studies we do use lung injury scores, depending on what the research protocol suggest we use. If you do observations on our staff, they will look at those parameters that you've listed and make a clinical diagnosis.'

'On a clinical basis, the problem with that is the criteria are so tight that you would treat very few people and probably more in reality than that have a ventilator-associated pneumonia… It's good at benchmarking, if you like, to compare ICUs. It's not good for the individual patient because you need such strict criteria to make it that you would be under-treating.'

'We don't use a specific scoring system here.'

'…our practice is not to use a scoring system on the ward round to make the diagnosis… the ultimate question you're facing is not, "Is it a VAP?" But, "Should I start antibiotics for a VAP?" Which is actually – it sounds like semantics, but it's actually a slightly different question.'

'They are just in our mind, to be honest.'

'we have got hospital antibiotic policies. So, I'm sure we have. Yes, we have a policy for ventilator-associated pneumonia, in terms of antibiotic therapy, but the reality is that if somebody develops a ventilator-associated pneumonia we have a very detailed discussion with our microbiologists.'

'…there are other reasons that somebody could- oxygenation could be deteriorating that's not due to a ventilator-associated pneumonia. For example, they could have a lung collapse. That often happens when you're on a ventilator, depending upon how you're ventilated. They could have got what is called a pneumothorax, which is air around your lung. That sometimes happens when people are sick. So, somebody getting worse is not necessarily a ventilator-associated pneumonia… So, one of the most common conditions in somebody who comes into intensive care is that they are septic, and people who are septic, one of the things that they can get are leaky lungs and you can get infiltrates in the lungs. So, this is called acute lung injury. So, I mean, there are other causes of infiltrates in your chest x-ray, but somebody who has got new infiltrates, who starts getting a dirty speck coming up their breathing tube, whose white cell count is going up, would make us suspicious that they've got a pneumonia. '

'Well, in major trauma patients they can have purulent sputum because they're coughing up blood, or aspiration, and they could have all the other things as part of non-infective lung injury. It's not a very clean population and, equally, patients with chronic lung disease, fibrotic lung disease, cystic fibrosis patients, they will often have purulent sputum as part of their normal clinical state and it's quite difficult to differentiate new breakthrough septic shock or sepsis from their background. So there are quite a good bundle of things that would, if you do the fuzzy logic, I suppose, that would make you think of VAP but it's not perfect in terms of sensitivity and specificity. Those two examples being examples of where they might mislead you.

''Well, therein lies the problem, is that all the things I've talked about really are quite non-specific. Sputum load. Even that is non-specific really… There could be a source of infection somewhere else. Obviously, depending on what brought the patient in, but it could be their abdominal source, if they've come in with a perforated bowel or something. It could be their urine. There are lots of sites of infection. It could be their skin. It could be their lines. There are lots of other sites of infection and all those tests are a bit non-specific. So it could be somewhere else. And it could just be that the reason their oxygenation has deteriorated is because their septic process is worsening. That's the problem why oxygenation isn't specific. So you could get a worsening of the chest X-ray but it not be related to infection in the lungs. It could be related to ARDS or a condition that is mimicking an infection in the lungs but isn't an infection in the lungs… I think even the sputum change. Patient's sputum will change day to day. It doesn't necessarily mean that they're infected. And everything else, the things you've got down on your list there, temperature, white cell count change, oxygenation change… and change in the chest X-ray, could all be related to a system that's not related to lungs. If you've got pancreatitis, another condition that's driving your ARDS, then your chest may get worse but it's not related to your lungs.'

'In and of itself worsening [hemo]dynamic status wouldn't be particularly specific of VAP. It could obviously be any infection… High and low temperature could easily be sepsis of any source, not just VAP. It could also be drug reactions or neurological causes. Brain injuries can cause pyrexia. For example, high and low white cell count, again, could be either from just [marrow] suppression to low white cell count or Gram-negative sepsis which can be actually ventilator-associated pneumonia but is also highly associated with abdominal and urinary infections. High white cell count could be secondary to steroid use, which is quite common in the ICU. [Purulent] sputum is a classic sign but I suppose it could just be someone with chronic lung disease. They can have permanent sputum as a chronic thing… Worsening oxygenation could be ARDS fluid overload, heart failure, and many other causes. Worsening [hemo]dynamic status could be either cardiac causes or any other cause of sepsis. Infiltrations on the x-ray could be collapse from ventilation rather than infection. All

these things are consistent with VAP… I think certainly as individual signs they have limited sensitivity and specificity. You're going to have a lot of false positives and false negatives if you rely on [clinical signs].'

'It could be just the presentation of a primary pneumonia that's not ventilator-associated, for a start. So, you know, patients come in with sepsis or respiratory failure, and sometimes, it's not clear what's going on for 48 hours, but they may have actually been brewing up an infection that's not clear on the chest X-ray and gets worse when you resuscitate them in critical care. But, what else? I mean, the whole thing is together and all those things are very suggestive of infection. You're obviously looking at other causes of respiratory failure. Is it heart failure? You could get different traces from the chest X-ray compared with baseline. It could be heart failure. Have they got an underlying viral pneumonia, which often gets worse when you intubate and resuscitate patients? Is the sepsis another cause? You know, when they have COPD anyway, so they've got purulent sputum and they've got systemic sepsis of another cause which would cause all those other things. So, it's not necessarily VAP, even though you've got all of those clinical features. It would give you a suspicion of that, that's what we're suggesting.'

'We'd think about ARDS in a ventilated patient which could give very similar features. Think about cardiogenic pulmonary oedema, could give you a bilateral pulmonary infiltrate. Extra-pulmonary sepsis. Clearly you could get a patient who looked very like they've got a VAP but have deteriorated from bacteraemia from another source and got lung injury complicating that. Yes, there are differentials. It's sometimes tricky to know. You have ward rounds where people um and ah about whether we think we've crossed the threshold for a VAP or not.'

'Yes, exactly, so it might be a line infection, or it might be a urine or abdominal infection.'

'So, it's partly, it is a clinical diagnosis. If you get several of those features together in somebody… one of the things that would definitely confirm it is if you look a sputum sample and it suddenly started growing organisms in the spit, which you weren't getting before then, yes, you've got pneumonia, or if you start growing the same bugs in your spit that you get in the blood then, yes, you've definitely got some pneumonia.'

''We may just get deep suction aspiration, but we may bronch them depending on whether… We don't tend to bronch for diagnostic, only for therapeutic interventions here. So, if they've got obvious consolidation and lung collapse, we would bronch to try and improve that, and then, of course, you would do blood cultures, as well, which is another way of picking it up. So, that's the way we look at the microbiology… I know it's common practice to do bronchoalveolar lavage with a bronchoscope to try and get samples for microbiology. My experience of that is, again, it's a lot of pain for little reward. In other words, the microbiology we get is often unhelpful, if at all. So, for therapeutic… So if, for instance, they've got significant… if their gas exchange has deteriorated and when you look at the chest X-ray or examine, they're not ventilating a lung or a part of a lung, then it may well be because the airways to that lung are obstructed with secretions. We will bronchoscope then to try and wash out and suction the secretions to improve the gas exchange, and at the same time, obviously take samples. So, our indications tend to be more for therapeutic, as I said, for those reasons, rather than for diagnostic.'

''I think if somebody is a more common category of patient, I suppose, a general medical patient, a patient who's probably come in with actually community acquired pneumonia, a patient with COPD, a patient with another critical illness like heart failure, cardiogenic shock who develops features suggestive of a VAP, probably we would just take sputum samples or upper airway samples initially.'

'We have surveillance, non-directed lavage, on an alternate daily basis on the intensive care unit, performed by our physiotherapists currently.'

'…two to three times a week, providing the patient is fit enough for it. We do it as surveillance screening for respiratory infections for the intubated patients on the unit. Our physiotherapists are trained in doing that. We would instil 10ml, 15ml of saline down the endotracheal tube, leave a short dwell time of a few seconds, and then aspirate into a trap, and then send that off to be analysed in microbiology. Are you relatively confident with the results from that technique? From the evidence that we had… aspirations from supraglottic suction above endotracheal tubes and NBL samples, and occasions when they've done full bronchoscopic lavage samples, organisms are quite similar in that sort of situation. So, I think, from that point of view, if you get a positive culture from it, I think it is fairly specific in that situation. However, it may not be that sensitive of actually picking things up, because it is actually quite a small volume… there may be some false negatives with it, but I personally think it is better than just doing endotracheal aspirates. And not as comprehensive as the BAL, would you say, or is it…? No. No, it's not as comprehensive as the BAL. So, what motivates you to go that extra step and do a full bronchoscope lavage? …we would tend to do that when we're not clinically convinced that we have necessarily an infective process going on… if we have a suspicion, as I said, there is a tendency for the clinicians then just to start empiric antibiotics. If there is doubt there in that situation, and yet there is clinical deterioration, that's when we would do a full bronchoscopic lavage, but we are certainly not doing it on every patient with our suspected VAP, at the moment. Perhaps we should. Perhaps that should be in there, in current guidelines. I think there's evidence to suggest it should, but we're not doing it at the moment.'

'Yes. We do that [Mini BAL]. What that tells us is if there's something [growing], but of course that doesn't tell you that that's what's causing the problem. The reason we do them primarily is so that if a patient's chest does deteriorate, and we think they've got a VAP, we know what their microbiome is like, what they're growing in their chest. So that if we know that they've got [?] or something, and we know that that's sensitive to co-amoxiclav, then that would be the right drug to use… It doesn't rule-in or rule-out. It just tells us what treatment might be useful. We try and use it as a surveillance test, not a diagnostic test.'

''I think the relative advantages are it's very easy to do. I think it's ease of use. All you need is a suction catheter, a syringe, some normal saline, some sterile gloves and if you can feed a suction catheter down an endotracheal

tube you can do an m-BAL. It's easy to do.'

''[even with a catheter there is a loss of PEEP? I assumed it was the bronchoscope that caused…] No. There is, it depends a little bit. There are different ways of doing it. The simplest way to do it is just to pull the circuit apart and put the suction catheter with the syringe on the end of it down the endotracheal tube. You can put a port, a cap on the end. You can put a swivel connector in and open the port there as if you're putting a bronchoscope in and put the suction catheter down there and do it that way. Some people would do it that way if they were concerned about loss of PEEP. It sometimes gets a bit tricky and then you're fighting to get the suction catheter through the port. A bronchoscope is much more rigid to put through down a suction catheter. The way I grew up doing them was simply that you just disconnect the circuit, go straight down as far as you can down the endotracheal tube and then reconnect. Depending on how people do it, you can de-recruit them.'

'Right, well the benefits are you can get a decent specimen from which if you grow any microorganisms, you can be very sure of their significance. As opposed to organisms that just come up with a bit of sputum on a tracheal aspirate. Because you've got specimens from what should be a sterile site. Any bugs you grow from a sterile site are much more likely to be significant. There's your big advantage… I think frequently we'll grow something that we weren't suspecting and therefore that modifies the treatment. Sometimes the bronchoscopy may be negative and that might modify the treatment in so far as not starting antibiotics, or stopping antibiotics, or so on. Yes, it frequently has an impact on the patient management. Obviously if it didn't, we wouldn't do them, I guess.'

'We do do BALs. We don't do as many, I would say, as some other units. We tend to, say, somebody, if we're not clearing up spit and we wanted to get a deeper sample then we would do a BAL, or we would do a BAL if we suspected a particular organism. So, for example, pneumocystis is better to get a sample deeper down, which you get better from a BAL, but we're not, ourselves, a unit that does many BALs I would say, to be honest… about 70% of our admissions on ventilators… I would say about maybe 5% or 10% of our patients gets a BAL. So, I would reckon maybe one or two a week, maybe, at most.'

'I don't think we have a good rule-in, rule-out test for VAP. That's one of the problems…So what quite often happens… people start antibiotics anyway… We occasionally, but not as a routine, do a formal bronchoscopically assisted lavage. But then our microbiology labs don't provide us with a quantitative sample. It tends to just be a qualitative thing. So again, it would help us in terms of telling us what organism is there but wouldn't tell us whether this was truly a VAP or not. So I think we lack the diagnostic infrastructure to be able to rule-out a VAP… for the diagnosis of VAP I would say it was not very common. In terms of how many patients, we do NBLs, like I say, routinely. We've got that surveillance data. But to do a specific targeted BAL, done by one of the clinicians, I would say was pretty unusual. Maybe only once a month, something like that. Twelve a year. Some of my colleagues are a little more keen than others. Some less so. We've got a difficult weaning service at Wythenshawe. So sometimes we might do them in those particular patients, because somebody has already failed to wean them, and sometimes we think, "Have they got something?" So we would use it for looking for unusual organisms, or something that's very resistant, but we don't do it routinely to diagnose VAP. There is talk about us doing it more routinely, but our current practice is that we don't.'

'It's obviously invasive for the patient. There's a cost associated with it, in terms of time and equipment. If you do it properly you need to irrigate reasonable amounts of fluid into the lungs…There's quite often a probably temporary dip in their oxygenation associated with that. So some of our really sick patients, who are already on the edge respiratory wise, you're cautious about doing it, just because the window and the deterioration you might induce might be too much for the patient to handle… I think that puts people off. The time, cost, and the risk to the patient I think is what puts people off… I've always felt that the diagnostic yield from it is not great. You do the procedure, you put the patient at risk, [it takes time], and you don't gain that much more information from it. And, unfortunately, it's too easy to just start a patient on antibiotics empirically rather than with a diagnosis.'

'Along with the blood cultures. We do occasionally do BALs. We're not as heavy a user of them… for various reasons. In certain circumstances we do use them… They tend to be particularly if they are immune-suppressed and we're worried about PCP, which you require a BAL sample for or if we've got a positive sample but we really think it's a contaminant. Also, if there's a collapse and we're going down to try and open up the airways anyway, [it could be done] in those circumstances… [on PCP] I think it's quite relevant now. It's got a new name but not everybody uses it. It's classically in HIV patients with low CD4 counts, high viral loads, so probably treated [in compliance] with the medication. It can also be in any severe immuno-suppression type patients.'

'We would only then do bronchoscopy if there was a mechanical reason, like a therapeutic reason, or we were treating with antibiotics and the patient was getting worse not better.'

'[Using BALS on all suspected VAP?] I think we probably would. The service here are going more in that direction, trying to protocolise, and we are trying to become more alive to the downside of antibiotics, particularly the wrong antimicrobial treatment and unnecessary antibiotics, a quantitative approach to VAP treatment. I suspect when reusable fiberscopes come into play, maybe in the future if they get cheaper, then we probably will go down that- we certainly do a lot more bronchoscopy than we used to do, but that's partly because we're a lung injury centre so we have ECMO and that kind of thing. We would do more now because our consultant group are more expert at bronchoscopy.'

'[I] think if we had got a patient that wasn't resolving, and we weren't sure of what the diagnosis was, so a patient that maybe was a more long-stay patient, more likely to have resistant organisms… or viral stuff, then we would say, "Right, okay, we need to look a bit more seriously at this." And that's when we would do one. But in the routine chest deterioration we just don't do them, no. It does differ between clinicians…'

'We would do bronchoscopically-guided. The reason we might do that is if we had a focal abnormality on the

chest x-ray or CT that we wanted to guarantee we sampled. If there was a possibility of unusual pathogens, so if we were thinking about TB or aspergillus or something unusual, we would then want a bronchoscopically-guided BAL. Or if we're doing a high volume, if we wanted to get a high-volume BAL for certain unusual pathogens, then we'd do that. We probably do that – it's the kind of thing we might do once every couple of weeks to once a month. We're not a unit that broncs a lot of people in this way. I think we're a bit more conservative. We would tend to do it when we're thinking about immunosuppressed patients, or we've got a differential with [some non-infective things 0:15:44] or unusual pathogens and want the large volume.'

'talking about BALs, well, we don't do that many. Again, it's a fairly low risk but you are potentially disconnecting the patient from the ventilator temporarily and there is the risk of, certainly if someone is fairly sick, of the lungs de-recruiting. By that, I mean it's just that the lung bases are not being held open by the ventilator and they collapse down and, just, so your oxygen levels can be worse than they were before.'

'My feeling is, we probably don't do them enough, because they are time consuming. They are quite technical, in terms of deciding how much lavage fluid you need to put down. The potential for causing more clinical deterioration of the patient, the patient desaturating more.'

'I would say that, probably, I'm thinking about we do somewhere, and it varies per day, but we probably do somewhere between two and ten per day, I would say… That's across all of our units. We've got five big adult intensive care units.'

'Right. Well, if the story and the clinical findings are strongly suspicious of a ventilator associated pneumonia we might proceed to bronchoscopy at that point, to get some better specimens. We're not fans of diagnosing ventilator associated pneumonia basically from tracheal aspirates. They can be misleading. We might do bronchoscopy at that point. If the patient's too unstable or too unwell we might treat with antibiotics blindly anyway. That depends a little bit on the patient. It also might depend on what antibiotics they've already been on and how strongly suspicious we are that it's a ventilator associated pneumonia as opposed to something else… I guess the only relative contraindications would be a very unstable patient on a very high oxygen concentration. Or someone with a coagulopathy… There is often a group of patients where you really want to get some decent specimens and they might actually be not well enough to actually do so. That's the name of the game really.'

'It'll be fewer than one a day, but I guess – I'm just thinking that in the unit as a whole, it's probably two or three a week, I guess… [performing BALS] It will be medical staff within the unit. Sometimes consultants, sometimes trainee medical staff.'

'[BALS?] Yes, we do, but not at this point in the pathway… So either therapeutically, so if somebody, for example, comes in with a severe pneumonia, as evidenced by the clinical signs, so oxygenation and the radiological signs of a chest X-ray, then we'd be more likely. Again, it's not on the first day, but we'd be more likely to do a BAL, a bronchoalveolar lavage. So that's a… washout, but it's both diagnostic and therapeutic as well… I would guess once to twice a week, so 5 to 10 per month'

'That will be entirely context dependent. So, if we had a transplant patient, or a stem cell patient, or a patient with a complex history and likely upper respiratory colonisation, then we would have a lower threshold if we're doing a directed bronchoscopy to take brush samples or selective samples. If it was a more standard patient, we would probably be pragmatic. If we look at 100 patients, we can't use bronchoscopy in all of them. We would probably take sputum samples from their airway but be alive to the fact that if it grows staph aureus or yeast, then that is probably colonisation rather than infection. We would only then do bronchoscopy if there was a mechanical reason, like a therapeutic reason, or we were treating with antibiotics and the patient was getting worse not better.'

'I think it's more a logistical limitation from the technology. We have video bronchoscopy, but we can't do a video bronchoscope on 100 patients. They would get immediate bronchoscopy and protected sampling if they were an ECMO patient or if they were a transplant patient, or whether there was a complex microbiological history or there was some issue that made diagnosis imperative. For example, if they were already on broad-spectrum agents and we wanted to direct against a particular organism or we weren't clear what the likely organism is, or maybe there is a patient who is intubated with cystic fibrosis, that kind of thing, where we're trying to work out what the pseudomonas resistant pattern was, then we would jump straight in. There isn't really a rule, it's definitely decided based on a mixture of staff experience, availability, patient context, category and response to treatment.'

'So, we do them if we feel there is a real strong clinical indication to exclude a VAP, but if we think there's enough clinical information based on what we've got at the moment with the other parameters and suspicion of VAP, we would probably treat empirically.'

'So essentially we would potentially this on any. We might have a lower threshold for certain groups of patients, particularly those that are immunosuppressed. So patients on-Cancer, chemotherapy or haematology, chemotherapy. And sometimes that's led by the microbiologists who will say a BAL sample will be much more useful to us than a normal sputum sample.'

'I think it is around the logistics. I think there's an element there of it being invasive. I think there's an element of it being time consuming. I think it is a technique that, to do it properly, is quite a skilled technique if you want to get a true alveolar sample. I think there are concerns about causing clinical deterioration, and there may be a situation whereby the clinician would feel their oxygen requirements, the pressures on the ventilator, are too high to say that it can be performed safely.'

'It's not completely risk free. Essentially, what happens is you have to either disconnect the patient from the ventilator or you can stick your scope down through a little special connector to suck the stuff out the lungs. If you put anything into the circuit, particularly if somebody is very sick, then they can lose some of the pressure that's holding the lung bases open. So, there is a little bit of a risk of your lung bases collapsing down when you're doing that. So, it's not risk free

but I would say the risk on a BAL is fairly low, to be honest.'

'I think people with critical oxygenation. I mean, BALs have repeatedly been shown to improve the accuracy of your diagnosis, with regards to whether it's a contaminant or not. They've never been shown to either reduce ventilator length of stay or mortality. That's the reason we don't use them as much because at least transiently, they can worsen oxygenation and [if you are] really critical oxygenating, it can be really quite dangerous. We wouldn't used them almost ever in a critically oxygenating patient unless there was a therapeutic benefit to try and improve that oxygenation but not for diagnostic purposes… Everyone that's intubated in ICU, excluding the ones purely for airway protection, will have significant gas exchange abnormalities, otherwise they wouldn't have needed intubating in the first place. I suppose [when an ICU patient is critically oxygenating] – you're talking about people on 100% oxygen and paralysed with medical agents to do that... When I say critically, it's quite a small percentage of the total number who are intubated.'

'Your disadvantages are it's a sort of invasive procedure. You may make the patient a bit unstable. It will interrupt with their ventilation. They'll need more sedation or paralysis if they weren't already. There's a potential risk of trauma, tube displacement. Their oxygenation may deteriorate as a result of the procedure… Not all bronchoscopies are, not all BALs are the same quality. Clearly a negative one doesn't necessarily mean there are no bugs there, it just means you didn't find any. It may be because previous antibiotic therapies made it difficult to culture anything. A positive one that's heavily contaminated with other [emphysemial ?] cells so we know that it's not a good specimen may make me less likely to treat.'

'I think there are concerns about causing clinical deterioration, and there may be a situation whereby the clinician would feel their oxygen requirements, the pressures on the ventilator, are too high to say that it can be performed safely… where you've got patients on really high oxygen requirements, and I would say… I have quite a high threshold… from a respiratory background…over 80% oxygen, and requiring high amount of PEEP, over 10 of PEEP… Well, clearly, most of us would try nowadays to use just moderate airway pressures, but if they're on the higher end of airway pressures on the ventilator, I would have some concern about causing further desaturation. Because, in that situation, you are then going to be in the realms of potentially trying to do alveolar recruitment to improve oxygenation post-procedure, and whether you can rescue them if they desaturate, that's the concern.'

'If somebody was on very, very high amounts of oxygen or if somebody was requiring a lot of pressure from the ventilator to keep their lungs open, then I probably wouldn't do it. I would just treat them empirically with antibiotics, rather than actually exposing them to the risk of that.'

'There will be. Obviously, but they're not that common. I mean, you wouldn't want to stick a bronchoscope into someone who has got a hole in one of the bronchi, you know, post-surgery, or something like that, or somebody who has had…We do a lot of thoracic surgery, so we've got to be slightly careful in thoracics that you don't stick a bronchoscope through a sutured up bronchus, or something like that. But then, I mean, it's relative contraindications; it's more of an absolute contraindications. Clearly, if the patient's, I guess, gas exchanges is so borderline that when you start to intervene you could push them off the edge, they're another contraindication, but it's all relative. But there aren't that many absolute contraindications to bronchoscopy.'

'…patients who it's absolutely contraindicated. I think if you've got a patient who's very unstable and PEEP dependent and you're really struggling with them, unless a bronchoscopic BAL is seen as being a diagnostic and therapeutic intervention – so you've got an area of lung that you might be able to wash out and re-recruit, then you would probably say, "I'm at risk of destabilising this patient. The length of time it would take me to get the scope in." If they're hypercapnic and you're worried about CO2 clearance because CO2 clearance can be an issue. They're the kind of ones where I wouldn't want to do it.'

'So the other patient groups we would be worried about are those who have poor lung compliance and who are already requiring high ventilated pressures. So, they would be a group I would definitely avoid as well.'

'The patients we wouldn't do, it would only be contraindicated in- Well, the main contraindication would be patients who we thought wouldn't tolerate the procedure, so with severe refractory hypoxaemia.'

'anybody with severe underlying lung disease, both obstructive and restrictive interstitial lung disease, etc., may preclude it. Actually, I take more into consideration the physiological parameters at the time, the physiological state of the patient at the time, rather than the diagnosis.'

'Part of the problem with BALs is you're still taking a microbiological sample which would take, at best, 24 hours, if not 48 to 72 hours to give you an answer.'

'It's not great actually here. We've suffered from a problem with centralisation of pathology services. I don't know if other people have said this but there's been a driver… It's not infrequent for there to be a really long delay of days and days and days before knowing things. Sometimes we get some things back quickly. We can send stuff off urgently by courier and sometimes have a quick look. We find ourselves more often than not waiting longer than we think we should for m-BAL results. If we want anything else like unusual stuff; galactomannans or other bits and bobs, they can take an absolute age, so not good.'

'Realistically, we can get provisional results in about- Well, 48 hours… We get a qualitative result after that… And then the full result, including sensitivities, etc., will probably take another day, so 72 hours.'

'You might get a gram stain. We might get stuff out of blood cultures, but usually nothing useful for 24 hours, and certainly mostly 48 hours before we have anything that may be of any help… the gram stain can be useful if it's done, because it can tell us whether it's gram-negative or gram-positive. So, if it's gram-negative, then we'll obviously treat probably with Gentamicin. If it's gram-positive, then at that stage, we might stop the Gentamicin, or if it's just gram-negative, we might just stop the Tavacin, and just carry on with the Gentamicin.'

‘Yes. If we had no idea initially what we were treating, and obviously we wouldn’t have because your BAL takes a while to get any results from, we would cover gram-positive and gram-negative with antibiotics that we thought were most likely to work given those possibilities. Broadly speaking, we would give broad spectrum cover and then if we get results subsequently, we would then revisit what our antibiotics were and then deescalate, as the term is… If you ask the lab to do a gram stain, you’ll get a result back in an hour or two… That’s only going to give you a ballpark idea of what’s growing, but that can be useful. If you are awaiting cultures, then classically we don’t’ get a report for 48 hours. However, if there’s a very heavy growth within a shorter timeframe, then we would normally get a phone call from medical microbiology to inform us of that.’

‘[What proportion of patients that have come to the ICU are already on antibiotics?] The answer is I don’t know, but if I had to guess I would imagine it’s reasonably high because patients don’t usually come straight to intensive care, who are very sick. We would usually pick up patients from a ward, or the patient will have come through the emergency department, and if they’re unwell they will usually have had some, either, oral antibiotics or if it’s A&E they might have had a shot of intravenous antibiotics.’

‘only about half the time you get any valuable microbiology from cultures.’

‘I would usually start them if there’s good evidence of clinical deterioration, and I’ve got a strong clinical suspicion that there is a respiratory source. So, that I would have new changes on the chest X-ray, compared to the X-ray performed on presentation that I think is compatible with consolidation and the physiological deterioration, particularly in worsening oxygen, and presumably change in sputum, or indeed if I had had a positive n-BAL come from our surveillance screen. A surveillance screen with a positive n-BAL wouldn’t necessarily make me say that I would have to treat the patient with antibiotics for that organism that had been identified, unless there was evidence of clinical deterioration, and clinical evidence of the pneumonia.’

‘My concern there is that often the factors that we’re taking into consideration, as I’ve highlighted, are very, very non-specific, and, are we really starting antibiotics based on the toss of a coin…

‘I think the overall clinical deteriorating position of the patient really drives you to do something, and that’s nearly always starting antibiotics. If you think that their chest is deteriorating quickly then you think, “I will start some antibiotics for it”. Again, in a patient that maybe is weaning from ventilation and is doing okay and then deteriorates again, that suddenness in the change of the condition, in their respiratory condition, is something that pushes us towards thinking, “Right, I will start some antibiotics.”

‘It is, unfortunately, a judgement call. Definitely when the patient is [clinically] deteriorating, so oxygenation is getting worse, particularly if they are on a worsening inflammatory response to that [?] – either rising white cells or CRP, which are the other blood tests as inflammatory markers that we use. Particularly if [new] infiltrates were consistent with infection. I think that would be the most confident time I would start antibiotics. I suppose the other time when you are maybe less confident that it is an infection but you’re more… It would be when they are clinically deteriorating. You’re not sure it’s infection but you don’t want to miss it because they are getting so much worse that there isn’t any room for manoeuvre if you make a mistake.’

‘It would be a general deterioration of the patient. It might be rising oxygen requirements along with a temperature. A change in the x-ray. A change in the white cell count. Clinical signs of infection on clinical examination. Any of the above. Any of those, or a combination of.’

‘we do not have a lot of multiresistant bugs here… Most patients with multiresistant organs, we guess, have been imported from elsewhere. So, repatriations from the neurosurgical cardiac unit. So, most, you know, even hospital-acquired pneumonias, are not multiresistant. So, we would start empirically, if we’re going to. Often, we don’t treat straightaway. If the patients are stable we might hold off antibiotics and see what happens. Given, as I said, that the diagnostic signs of nosocomial pneumonia are actually not particularly specific. So, there’s no absolute… We would not always start antibiotics straightaway. If there was a deterioration in their overall clinical condition, we would, but if it’s just a chest thing, you know, a bit of a temperature, a bit more purulent sputum, gas exchange is not too bad, we probably wouldn’t start antibiotics. If they’re obviously unstable and they’ve got an obvious new pneumonia on a chest X-ray we would, but sometimes you can get those carrying a pneumonia without those last two signs. So, yes, we would start empirically. We usually start here now with Tavacin, which is mainly… Sometimes, if they haven’t had antibiotics prior to that, Co-amoxiclav, but Tavacin is probably our main first line, you know, broad-spectrum antibiotic, empirical antibiotic. If we think there’s a risk of gram-negative, then they would have some Gentamicin, as well, probably. If they were penicillin allergic, they would get Vancomycin.’

‘I think the key drivers in my mind are when several things align to tell you that it’s a VAP. If you’ve just got a fever, or just got a white count, or just got a change in sputum or just got those things, then I wouldn’t. Equally, if I’ve got a bit of a change on my chest x-ray that could be an early infiltrate, but the patient’s condition hasn’t changed in any way and there’s no increase in the FiO2 then I wouldn’t. The people I would are the ones where there’s been a change in the patient’s condition. Maybe there’s been a fever overnight and, or the white count has gone up this morning. The oxygen requirement is slightly increased and there’s an infiltrate. I would probably do it in the absence of a new infiltrate if I’ve got lots of markers of respiratory deterioration, so increasing FiO2, purulent sputum and a fever. I wouldn’t say, “There’s not yet any infiltrate so I can’t possibly start anything.” I would probably start something because I’d say, “Why wait for the problem to deteriorate further?” If I’ve got an infiltrate and some other sign of a VAP, I would start. That would also depend a little bit on the patient. I can think of a chap I saw recently where we were all quite nervous about him getting another VAP because he was in a weaning phase of his illness. But he already had an organism in his surveillance BAL that was multi-resistant, and we wanted to be careful about making a good judgement call and not using up all the antibiotics we had available. There are patient specific factors that might

make us say, “Let’s just wait. We need a higher threshold for doing this, or a lower threshold.”.’

‘We would usually start treatment as soon as the suspicion, the clinical suspicion, arose, and at whatever time of day that was… we don't wait for the cultures to start treatment.’

‘So, again, it’s a clinical judgement, but if somebody is sick and we think they’ve got a ventilator-associated pneumonia then we will start the broad-spectrum antibiotics. If we’re lucky enough to, once we’ve started the antibiotics, like, say, the microbiologist will just say, “Look, this patient has got pseudomonas, or something, in their spit,” then we can narrow down the spectrum, or if they’ve got a, I don’t know, a strep pneumo or something. So, basically, yes. We start broad, and if we’re lucky enough an answer from microbiology then what we do is we tend to de-escalate.’

‘We’d certainly use the most recent microbiological isolates to tell us what antibiotics it would be reasonable to start if we want to treat for a VAP. I don’t think we would be swayed. If we’ve got an organism but there are no other features, we would usually sit tight and acknowledge the limitations of that. It would factor in. The main way in which I think I use it and others use it is to say, “Okay, I think there’s now a VAP.” If I’ve got an unpleasant multi-resistant gram-negative organism in my m-BAL then I would probably start with something that is going to cover that. Whereas if I haven’t, I would maybe start with a different antibiotic. It would factor into the decision about which therapy to start.’

‘There’s a group of patients where I sense that in terms of getting results, we say, “The stakes are quite high here.” If you’ve got a patient with multi-morbidity or who’s quite frail and you think they’ve got one shot at getting out of ICU, or a high-risk post-operative surgical patient who’s still ventilated. I think I certainly would, and most of my colleagues would probably say, “Look, we want to treat this sooner rather than later. If we get this wrong and we delay, and we wait until the VAP’s developed and then there’s multiorgan failure from a VAP, that would be a real shame.” Then there’ll be other patients where you’ll say, “Well, they’re fit and young. They’ve been intubated for airway protection. There’s no good evidence yet so we can afford to wait.”.’

‘…the incidence of Gram-positives and Gram-negatives changes as time goes on. A lot of hospital acquired infections tend to be more Gram-negative organisms, although we’re seeing more of a resurgence of Gram-positive organisms. So, if somebody, we think they’ve got a ventilator-associated pneumonia, we would probably, actually, empirically treat quite broadly because, often, when you’re treating somebody for a ventilator-associated pneumonia, you have no idea what the organism is.That is one of the problems in intensive care, is that the markers of what is the bug that’s causing the problem, a lot of the time they’ve maybe had previous antibiotics, or you just- I mean, our pick-up rate from blood cultures or sputum is actually not that high. If somebody is on a ventilator, and they’re unwell, then we tend to start broad and then reduce down, if that makes sense to you.’

‘We would tailor them if we got a result back, because we have a microbiologist and they would try and push us one way or the other. That’s not really protocolised, it’s a recommendation that we narrow the spectrum once we identify a causative pathogen. But often the patients remain on the empiric antibiotic combination. It’s very difficult, there’s no protocol around that, really… As a general principle, we would try and narrow the antibiotic profile of the patient where possible, but it’s highly dependent, again, on context, clinical course, responsiveness, etc.’

‘We would have daily microbiological ward rounds. So, if we hadn’t got an organism, we would probably go empiric; I think at the moment for us something like Co-amoxiclav, which is fairly broad spectrum, would probably be our first choice, but then we would actually tailor it based on advice from our microbiologist, what else was going on with the patient.’

‘In our hospital, for ventilator-associated pneumonia specifically, then we would have a Gram-positive and Gram-negative, yes. Then obviously tailor it further depending on the patient’s previous results and whether they’ve been in hospital for a very long time or whether they had previous antibiotics courses and whether they are immuno-suppressed, as well.’

‘…the evidence is that if you undertreat people they do badly, but if you overtreat with more antibiotics with a broader spectrum than they need, then you’re encouraging bacterial overgrowth and resistance. You don’t want to miss anything initially, but once you know what you’re treating, then you hone down to focus on that.’

‘Yes, I think it would be useful. If you could quickly know that what you’re dealing with is a pneumococcal pneumonia for example, and you want gram-positive cover for strep, there drug of choice there for us is benzylpenicillin if it’s a sensitive organism. That’s the best antibiotic. It actually works very well and it’s nice and narrow spectrum. That’s actually the best treatment for the patient. There, giving something very, very broad might actually be less effective for the patient. There’s an advantage to that in actually getting a better, narrower antibiotic. Also, avoiding the problems of resistance and avoiding some of the risks of c-diff and other colitis and all these other problems. The gram-negatives, yes again, if you know you’ve got one then our microbiologists would probably want us to use things like aztreonam or something as narrow as possible for our gram-negative organisms… If you don’t know, you end up going very broad.’

‘Right, okay. The first thing is, if we put them on empirical broad-spectrum cover, the thing that would make us stop some would be finding a specific organism, or organisms that we could narrow down the treatment of. Broadly speaking, say we would have started gram-positive and gram-negative cover, if the BAL shows only gram-positives growing, then we would probably stop the gram-negative cover and vice versa. That’s the first thing. The second thing would be, what you’re really asking me I think, is duration of treatment. That’s quite a difficult one because nobody really knows. I guess generally what we would do is we would probably say, “Let’s do a seven-day course and review.” Then if the patient is substantially better, we might stop at that point. If they’re not then – I guess if they’re not substantially better, that would provoke a, “Are we missing something?” sort of scenario. Or, do we need to change the antibiotics empirically and re-investigate and so on?’

‘What we would do is we would make an assessment as the most likely causative organisms. We actually have a fairly longstanding and frequently revised agreed guidelines with our microbiologists. That’s pretty heavily protocolised as a rule. There will be deviations from protocol based on; what exposure has the patient had to antibiotics? Are they immunosuppressed? We get a lot of patients who are immunosuppressed in our unit so that might influence things. Basically, it’s protocolised with intelligent alteration, if you like.’

‘For certain organisms we know really well… e-coli from urinary tract infection and things we are given data about what… Partly because the resistance is high in the community. They tend to feed back to us and say, “This is what we’re going to use.” We have an online formulary… We use MicroGuide, which is one of the apps. Microbiology inform pharmacy as to what to use as our empirical first line, and they do change that from time to time based on our background resistance rate… [Is it effective?] MicroGuide? It’s like any microbiological guideline. The advantage of it is because it’s electronic obviously if they get a change in what they want as the first line then they change it in that and it’s changed in all places simultaneously. Rather than printed or even electronic guidelines that are written at one time and then have to be updated on a cycle. It’s pretty live, in terms of what they change as the first line treatment. We just switched recently.’

‘Yes, we are. In a liver transplant patient, we’re much more likely to go to higher line antibiotics rather than the standard protocol. There’s an interesting effect, because I’m the informatic lead I can see antibiotic prescribing practices and on the transplant ICU, if they get a general patient, they are more likely to use second line antibiotic or third line antibiotic, antifungal and broad-spectrum agents in general ICU patients.’

‘The time in hospital over sort of five days, that may affect what antibiotics you’re using. I would correlate that with what we would do in hospital-acquired pneumonia, which I think this maybe where this has come from, because certainly our pulmonary in intensive care for hospital-acquired pneumonia, less than five days, we would use Co-amoxiclav. Over five days, we might use a quinolone, such as Levofloxacin, as a first line in that sort of situation. So, the duration in hospital probably does have an effect.’

‘What we will often do is if somebody deteriorates, we usually start fairly broad and what we do is we phone microbiology, or we look up on the computer system to see if they have had any resistant organisms in the past. In which case, we’ll start even broader than normal.’

‘[…at what point do you usually start to treat a suspected VAP?] Immediately, basically. We use the local protocol. So VAP will be treated with piperacillin/tazobactam and probably gentamicin. Is that empiric? That’s empiric, yes. If we saw a patient who had a VAP we would do a baseline sample and then immediately start antibiotics.’

‘If somebody had deteriorated a bit but I wasn’t that worried about them. So, say they were requiring 40% oxygen but their oxygen had gone up to 50% and they’d had one blip in their temperature, I might wait. They could possibly get a BAL and wait for a while. If somebody had deteriorated rapidly and their chest x-ray was much worse, and they had the markers of infection that we tend to look at, like white cell counts and CRP, if they had shot up dramatically, very quickly, then I might not wait for a BAL, I might just hit them with antibiotics straight away. It’s really a clinical judgement as to how sick I think they are at the time.’

‘[Do you value the narrowing of antibiotics?] Yes, definitely. The only problem is that… I try and minimise the spectrum of antibiotics whenever I can, based on the current microbiology, but the tendency is sometimes to go broad and stay broad. If the patient doesn’t get better they stay broad, and if the patient does get better some of my colleagues say, “I'm staying broad anyway, because they got better, if you know what I mean, so clearly it did the right thing.” That’s the challenge with antimicrobial treatment on ICU.’

‘…gram-positive and gram-negative…Is this useful information? I don’t know the answer to that actually, because I think quite often you will get mixed flora, and I think it depends on what the…This is where the importance of the underlying respiratory disease is with the patient. So, for example, if they’ve got a severe underlying respiratory disease and there’s been previous evidence of, for example, with COPD, of exacerbation with different organisms, where they’ll be gram-negative or gram-positive, that would probably make me tailor my antibiotics then more specifically to what I had identified. I think you might come down even further. The other sort of thing is whether, for example, patients with underlying lung disease, who may have bronchiectasis, or conditions like that, where they may previously have grown Pseudomonas, we would probably target antibiotics more specifically towards that group of organisms to start with.’

‘More effective antimicrobial killing, because there’s some antagonism between different antibiotics in terms of action of particular MICs. I think if you know that a bug like VAP is caused by a particular microbial organism, then it’s better to treat that with the most effective antibiotic. For example, flucloxacillin is probably a better antibiotic than vancomycin for treating sensitive staph aureus pneumonia, particularly if you give the right dose. So there is some sense in adjusting and, also, if you’re on more than one antibiotic unnecessarily, you’re exposing the patient to the toxicity profile of the inactive antibiotic unnecessarily. That is almost unethical, quite a dangerous thing to do, and it also conceals deterioration for other reasons, because you might put it down to the antibiotic, and you’re also exposing the patient to selection of organisms that are resistant, you’re denuding their gut bacterial flora which is probably an important part of the immune defence, and exposing them to the risk of further resistant hospital acquired sepsis going down the track and C-dif infection, and things like that. So there’s a whole range of reasons why it’s better to focus and use one effective antibiotic… And cost, as well. Antibiotics are very expensive, so in health economics then I think that’s an important thing to remember, too.’

‘Cheaper, better and safer.’

‘So, would there be an advantage to having Gram-positive and Gram-negative information? Yes. So, one of the biggest things that determines antibiotic resistance is, one, the dose of antibiotics and how broad-spectrum the antibiotics are. So, for example, if somebody deteriorates and you know what organism it is then you will use a much more narrow-spectrum

antibiotic, which will be as effective as the broad-spectrum antibiotic, but you won't necessarily have the potential risks of resistance that you would get by using very broad-spectrum antibiotics.'

'if a patient responds and is getting better then people go, "They got better with antibiotics. Let's carry them on." And if they don't respond, they're getting worse, then they still go, "Let's carry them on, because they've got worse." I think it's really difficult getting people to stop antibiotics. It is really difficult. Because we don't have a good rule-out test. And the risk to the individual patient of carrying on another two or three days… is just too low so people carry them on. I like to think that if all the microbiology turns out negative and the patient hasn't deteriorated that I will stop the antimicrobials after three days, but, like I say, hand on heart I'm not sure I even do that. I would like to think I do that, but I'm not sure I do.'

'Another example, which is sort of analogous, is interventions that are supposed to be adjuvants to treatment in sepsis. For example, if an intervention requires a physician to do something, like it requires a line to be put in or a consent to be taken or something that involves actual increased workflow, then the doctor is less likely to use that intervention as opposed to using hydrocortisone, steroids, because to use hydrocortisone as an adjuvant to treating septic shock, the doctor only has to prescribe it, which is a second's work. It doesn't require any work on their part, so they're more likely to do it… There are other factors, for example, in handovers. Say one consultant is on for a week and then they hand over to another consultant, they're very unlikely to stop, even if the plan is to stop antibiotics on the day of handover, they are likely to continue for a further 24 to 48 hours to confirm in their own mind the patient is clinically feasible to stop. When it comes down to it, when it comes to the decision on the ward round, there is a human factor of influence in that, as well. There's lots of very complex stuff that goes on in the background.'

'I think that you kind of go, "Well, I'm probably going to give the patient antibiotics anyway, so let's just do that," and to a certain extent the diagnosis becomes a little bit more academic. If you look at the number of patients who when we've looked at it you would confirm that they probably had got a VAP, versus the number of patients given antibiotics for a query VAP, then there's hardly any relationship. A huge number have query VAP but don't meet diagnostic criteria, if you were being strict about it. We probably over-treat a huge number of people, and that's detriment to the [managed microbial] use.'

'…this is the human factor thing. A doctor doesn't have to do any- it's literally a few seconds work for a doctor to prescribe or continue an antibiotic. To stop an antibiotic, though, it takes thought and processing time for that doctor, exposes them to a bit of stress because it's more difficult to stop than continue. So there's a lot of human factors involved in that decision making. If the doctor themselves had to give the antibiotic, then I think you'd get a lot more stopping of antibiotics.'

'…because the intervention that may be needed could cause potential clinical deterioration, and is clearly quite time consuming, and it's far easier just to give some antibiotics.'

'The oxygenation index or Pa/FiO2 ratio improving steadily, so a trajectory of improvement not just a static single number improvement. Improvement in ventilatory parameters, so lung compliance, more volume is going into the patient with less pressure. The patient systemically improving, for example, noradrenaline, which is a drug used to maintain blood pressure, that may start to reduce steadily again, not a single hour but a steady improvement. Improvement in numeric blood result, so recovery of neutral count, is it supressed or falling neutral count if it's raised. Recovering platelet count is a very powerful indicator of improvement, because that doesn't happen if the patient's not getting better. Falling C-reactive protein or, we don't use it, but procalcitonin would be another measure of improvement. But usually it's a global- it's obvious the patient's getting better globally. They become less problematic and they usually improve on all those fronts. There are usually multiple sources of evidence of responsiveness and improvement.'

'The obvious response to that, from a clinical point of view, is signs of clinical improvement. For example, if we go back to the clinical features and the suspicion; if the patient had got features of sepsis, and poor haemodynamic status, and that had stabilised, oxygen requirements coming down, the tracheal aspirate perhaps changing in colour from purulent more to mucoid. In that situation, we should then perhaps be thinking of stepping down and de-escalating. I think with de-escalation, there's a situation both of changing the route of administration of an antibiotic as a de-escalation. For example, going from intravenous through to enteral; that clearly has to take into consideration whether you're satisfied the patient is absorbing internally. Then you've also got the situation of, during the time in the investigations, have you had a more specific positive culture, if you've started with an empiric antibiotic that you can actually target? And I would bring that into the same bracket, as well; and if that is the case, I think it should be more focused to the organism.'

'Clinical improvement, alternative diagnosis that has been proven to be causing the deterioration and completing the course.'

'It's definitely a collection [of signs]. I think improvement in inflammatory markers, in particular, and resolution of pyrexia would be the two big things. Improvement in oxygenation, in particular, can lag behind. The patient is often… consolidated even once the infection is cleared in ICU. I'd want some improvement in the ventilator parameters.'

'Well, this is a very interesting question. Nobody actually knows how long you should give antibiotics for, for most conditions. Empirically, we tend to give five days, or seven days, of antibiotics, but nobody knows if you just give three big doses of antibiotics you kill the bacteria, per se, but then you're left with the consequences of the infection or the systemic upset. So, how long you give antibiotics for, there's not actually that much science behind it, but most people empirically give five or seven days of antibiotics, and the things that we're looking for are, like, your oxygen levels improving, the amount of support that the ventilator is having to give being reduced, the markers of infection falling, like, your white cell count or your C reactive protein. So, these are the things, but empirically, irrespective

of that, most people will get five days of antibiotics and then stop.'

'So our default is for five days of antibiotics, and, certainly if it was for a VAP, we would almost always complete that course… So in terms of whether we continue after five days, again, that's under guidance from the microbiologists. But after five days, if there has been no response or limited response, then we would usually change the antibiotic rather than stop them… it's holistic improvement in the clinical conditions. So absence of a temperature, resolving inflammatory markers such as white blood cell count, improving oxygenation and other aspects of the clinical conditions.'

'…we're very strict about how we manage our antibiotics. So, people get a five-day course of antibiotics, full stop. So, they'll get five days, and at the end of five days, we stop the antibiotics and review. Occasionally, we might do seven days, if they're complicated. The physicians and the surgeons would want the antibiotics to go on forever, but we're very strict about it. We'll have microbiologists, so we'll stop at five days and review, and usually, that's it. If they get unwell again in 48, 72 hours, then we may restart, but it's a five-day… I'm not personally too fussed about what's happening to all the other things that we talk about because you'll often see an elevated temperature, elevated inflammatory mediators, and indeed worst chests for a long time after a pneumonia. It takes a while to clear it, and that doesn't necessarily mean it's infection anymore.'

'I think we would, the first thing is when we start them, we would generally try to set a target for how long we think we think we'd treat for. Of course, we review it every day with a microbiologist. We would stop if we'd said at the beginning that we thought that we should do five days, or seven days, or whatever it is that we decided to do, which is often five days. We might then say, "Okay, we'll stop because we've reached our pre-defined endpoint." We would definitely stop sooner if we revisited the diagnosis the next day and decided we were wrong. For instance, if we started antibiotics and then we isolated an organism that we knew was not a cause of VAP but was causing a soft tissue infection somewhere else for example, we'd stop those antibiotics and tailor them for the other infection. We'd stop them if we were moving to a palliative mode of care, but that's probably not what you had in mind. We try to narrow them. If we got back an m-BAL that had [grade dependent five 0:28:57] ___ units of an organism that we thought was causative, then we would probably try to narrow things down if we hadn't isolated any other organisms. I suppose if the patient rapidly improved in three days and that was clinically with the decline in oxygen requirement, sputum getting better and inflammatory markers getting better, then we would probably say, "Okay, well three days may well be sufficient here. Do we really need to continue them if the patient's better?"

'We are trying to drive the human factor side of that a bit, so we are drying to mandate and governance that. When the prescriber has to- because we have an informatic system that probably in the future will mandate that they have to put an indication for the antibiotic and an intended duration, so the first step is that the prescriber has to place a duration on their prescription. Then within the informatic system are reminder flags that try and stop the antibiotics if you can do and then we have reinforcement from the microbiology service. Having said all of that, I think we probably still continue antibiotics for too long without any real clinical or scientific evidence base for more prolonged courses of antibiotics, which may well be more harmful than beneficial. But we are often painted into a corner, I suppose, where we've got very sick patients who have ongoing evidence of infection or are not getting better and there's no other option. So antibiotics are used as an only option, a magic treatment, because there's no other thing that we can think of to make the patient better'

'…there's actually no evidence that it's of any benefit for longer than that. I'm afraid you're treating yourself and doing nobody a favour if you carry on with that, antibiotics empirically, over and over again. It doesn't help the patient; all it does is encourage the growth and multiresistant bugs.'

'if somebody had previous antibiotics, that is definitely a factor.'

'antibiotics, yes. dialysis, yes. Immuno-suppression, yes... Previous microbiology, yes. I already mentioned that. Previously being going abroad, yes, particularly certain areas of the world where there is a lot of multi-drug-resistant bugs – southern Europe being one that springs to mind.'

'Prior use of antimicrobial therapy, yes. Because, clearly, if they've had your first line antibiotic very recently and they've come with another one, do you need to change it? Resistance patterns; that's where we would need advice from our microbiologists, in that situation.'

'If somebody has had multiple doses of antibiotics in the past, that is definitely a risk factor, or a lot of the patients that we get in intensive care have been in and out of hospital before, and so they may have picked up resistance. So, for example, somebody coming in who is, say, 25, who is unlucky and gets pneumonia, might be a totally different type of patient because they've not really been exposed to much, as opposed to somebody that has been bouncing in and out of hospital for years.'

'Okay, so time in hospital, yes. Prior use of antimicrobials, definitely. High prevalence of antibiotic resistance, yes… dialysis, immunosuppressive, previous microbiology… Yes, I would say they would all be considered. One of the things that we do, and I don't know if this is typical of all units, every single one of our admissions gets screened for colonising multi-resistant organisms. Again, that takes a couple of days perhaps to come back, but we would already know quite often if patients were colonised with multi-resistant organisms. That would influence our treatment.'

'Yes, clearly if they've had antibiotics. One of the problems we have, as I said, with patients coming back from elsewhere is they've often been given serial courses of antibiotics and are growing everything exotic that you could possibly think of… Immunosuppressive disease; well, you know, again, they would be on a different spectrum of antibiotics, probably staying broad-spectrum because you never know what you've got, you never grow anything. Obviously, previous microbiology has helped. One of the things we do look at when we start antibiotics is what might have grown in the previous week. So, if we're actually thinking, "This is just a contaminant, or just an overgrowth, not an infection," and they've become unwell, then obviously, we would use that microbiology when we start treating. Again, abroad, where

repatriations from elsewhere have clearly… you know, have multi-resistance.'

'Yes, [we see] quite a few patients on long-term antibiotic therapy. Dialysis. Immunosuppression. Previous microbiology. The hardest thing about previous microbiology is the lack of joined-up results in the NHS. Actually, finding out someone's previous microbiology. If they've been to an intensive care unit down the road six weeks ago, and then they've been discharged and then admitted to us, then it's difficult to know what their microbiological pattern was previously.'

'Long term antibiotics…absolutely. You can get all sorts of funny resistance.'

'So, time in the hospital… that's important because once you've been in hospital for a few days, as I say, organisms tend to be more Gram-negative than Gram-positive.'

'High prevalence of antibiotic resistance in the community or the hospital unit.'

'Previously, being abroad, yes and no. Again, if you've been on your holiday in Spain, just having been abroad, per se, won't make you at risk of something. If you have been, for four weeks, say, in a hospital in Spain where MRSA rates are much higher than this country.'

'…we're very nervous when we get patients repatriated from Oxford down to Reading because they've had a problem with candida auris. They've had a big problem with this multi-resistant yeast. Also, they've got a lot of carbapenemase producing enterococci up there. All our patients that come back from the unit actually from any external unit, we isolate in a side room and swab them all for several days before we let them out. Repatriations or transfers from other units is one of our flags, which you've sort of captured about hospitalisation.'

'I think your proximity to an airport does probably alter your flora and fauna a little bit.'

'Chronic dialysis patients. Yes. Particularly if it's peritoneal dialysis. They are more at risk.'

'…and the dialysis, and even a suppressive, absolutely. Because, this actually brings another realm of organisms that come into play if you've got evidence of good immunosuppression suppression for a prolonged period of time.'

'Immunosuppressive disease. Yes, 110% and, in fact, it's the immunosuppressed patients that we will often be more likely to do BALs on. So, by immunosuppressed, you could be immunosuppressed because of drugs that somebody is getting. So, for example, renal transplant patients, we get a lot of them and they're on heavy duty immunosuppressants. So, we will often BAL that population, or people who- we've got the, sort of, regional infectious diseases unit, so we get quite a lot of HIV patients, and they're immunosuppressed because of the HIV. So, these sorts of patients we would often BAL. So, I think that is definitely a risk factor.'

'…immunosuppressive disease and/or therapy, but tacked on to that you might include haematological malignancy or recent chemotherapy, because obviously that changes your risk of pathogens… we feed from the cancer hospital, so we've got quite a few patients knocking around with haematological malignancy and with solid organ malignancy who are on chemotherapy.'

'Immunosuppressive disease, yes, that would affect our thoughts. Previous microbiology, yes'

'All cystic fibrosis patients will be colonised with pseudomonas and they may have multiple strains of pseudomonas and they will have different resistance profiles. There are certain types of patient that are unlisted there that are at risk of resistant infection.

'Previous microbiology, actually, is probably the most important one in my mind, in terms of, it can give you the answer, and it's also very easy to find out by just pressing a button on a computer system.'

Theme 2: Current clinical need in VAP diagnostics

'I think the first area is accurate diagnosis, accurate and timely diagnosis in as non-invasive a way as possible, which makes it, you know, quite a difficult task … I think changing clinical practice, and having clinicians able to be confident in the tests that they are receiving in terms of the sensitivity and specificity, so it will change their practice to de-escalate… sometimes the results don't facilitate decision-making? Absolutely. I'm sure that happens. I suspect it happens both ways, but certainly I'm sure it happens in terms of the fact that, you know, you may get a negative microbiological result and you say, "Well, no. I'm going to carry on for another couple of days with antibiotics." Obviously, that has its knock-on effects with resistance patterns, and other nosocomial hospital-acquired infections, etc. So, it is an important one.'

'If I could wave a magic wand, I guess what I would like would be something that could give me with more certainty, the diagnosis of ventilator associated pneumonia and exclude other causes. Something that if it is a ventilator associated pneumonia, that could give me the causative organism much more quickly... Unless somebody comes up with a biochemical marker in the bloodstream that is highly sensitive and highly specific for VAP as opposed to anything else.'

'Well, I think the main problem is that it's common, and we're rubbish at it. That's the problem. As I said, mostly it's a diagnosis of suspicion. Very rarely do we confirm anything. We're treating for long periods of time with empirical broad-spectrum antibiotics, and the evidence… You know, there's very little rationalisation going on because it's not common to get, I guess, a pathogen in microbiology in the time frame that we're treating them to help us. So, I think, anything that could actually help us (a) decide whether they have got an infection, or not – so to stop treating in the first place, where it's just inflammation – and (b) to rationalise antibiotics early, would be of great benefit. That's my view on it, and I think most people would have the same view.'

'It would be nice if we had much quicker microbiological diagnosis, however that was achieved, by real-time rapid PCR. So near-patient testing that was reliable and easy to do, so that maybe we could target antibiotics a bit better than we do.'

So, often, blood cultures are negative. Often, sputum samples are negative. So, you think somebody has got pneumonia, they've got new changes in their chest x-ray, but you're not sure what the organism is. So, I mean, if there was something that could actually identify what the organism is, that would be very helpful.'

'So, I mean, obviously antibiotics, most antibiotics have got, like, Gram-positive and Gram-negative cover. Some antibiotics are very Gram-positive heavy. Some antibiotics are very Gram-negative heavy… It's more than just knowing what the organism is. It's more than just knowing whether it's Gram-positive or Gram-negative. It's actually knowing what the specific organism is would be far more useful.'

'[it] is pretty obvious when you come to prepare for intensive care exams at the end of your training – what you discover is that there is a lack of consensus on different ways of diagnosing VAP and what VAP is. There are various different – you know, the CDC definition and others for example, and different thresholds of picking organisms up on different ways of sampling. That's a bit of a cloudy issue. If you wanted to be a purist and say, "Well, a VAP is when there are definitely bacterial organisms in the lower respiratory tract where they should not be. They are there in sufficient numbers and they are to be pathogenic and causing an illness." Then what you'd need to know is what are those bugs? And what are they sensitive to? I suppose. At the moment, the problem is that we can tell you what we can grow from an endotracheal tube, but lots of stuff that's living in an endotracheal tube might not be causing a VAP. That's not useful. We know that's not useful because you have people who grow things in their m-BAL the whole time but never get a VAP. You get people that don't grow anything in their m-BAL who've obviously got a VAP. It would be very, very helpful to be able to make a more rational decision about antibiotic therapy early. Give it as narrow as possible as early as possible and have some sort of marker for – a hard marker for treatment cessation.'

'I think we treat a lot of patients for VAP who almost certainly don't have it. That doesn't mean to say they haven't got infection in another site of course. So, the antibiotics might have been the right treatment, just for the wrong diagnosis. There's definitely an over-treatment. So it would be great to have a rule-out test, although I appreciate rule-out tests are actually quite difficult.'

'So, I would say something to assist with the human factors and the unifying and strengthening decision making. That might include better awareness of the toxicity of antibiotics, which is vastly under-appreciated, particularly in junior doctors who think that it's completely benign to start a patient on antibiotics, where they could be lethal. I think that something to push doctors in that direction would be good.'

Theme 3: The potential value and role of the OMA platform

‘I think it would. It probably would allow you to narrow an antibiotic spectrum and to limit toxicity or exposure of antibiotics if you’ve got a very clear signal as to whether the causative microbial agent was Gram-positive or Gram-negative.’

‘Potentially, yes. [If there’s lots of bacteria down there] I guess that’s going to push you towards active treatment. Would the absence of bacteria stop you treating? I don’t think so, because you're at a single site, a small place, and infection can be very patchy. As opposed to a washing, where you get into my whole left lower lobe of the lung, this is just a tiny area. If you punctured the wrong bit.’

‘I think so. There are a few areas, I suppose. One would be if we’re trying to differentiate between bacterial infection and a collapsed consolidation non-pulmonary – that kind of thing where you don’t necessarily need antibiotics, at least not ones that cover the lungs. The second area is to differentiate between Gram-positive and Gram-negative infections and therefore tailor your antibiotic more specifically.’

‘To show gram-negative grows bacteria, which presumably is put into this explanted lung. Is there one to show up gram-positive bacteria? I mean, what we’re trying to decide here, aren’t we, is whether or not (a) there’s infection here, and (b) if we’re showing bacteria, are they significant? In other words, are they just what you would expect to find anyway? Are they contaminants? And what sorts of bacteria they are. So, if we can suggest that this technology was showing us only significant bacteria that was not part of a normal…what you would find, and I guess the alveolar is supposed to be sterile normally, or that area is supposed to be sterile. If we can differentiate gram-positive and gram-negative, then it obviously would help in our initial selection of microbiological therapy. The difficulty I also have is aspirating fluid up a long, fine piece of tubing. I am not convinced that’s going to work very well, personally.’

‘Yes, so if it allowed targeting of antibiotics in patients with- Well, any pneumonia, it doesn't have to be VAP. Could be a community-acquired pneumonia. If antibiotics according to gram-positive, gram-negative bacteria, I think that would be helpful… earlier and more accurate diagnosis.’

‘I think if this technology worked in a clinical environment; it does require bronchoscopies, as we were talking about earlier, but if this can help to detect organisms at an early stage, I think, yes, it could be practically very useful.’

‘I think it is something that would get taken up, especially if you were trying to use it, for example, in terms of early detection. I think the risks in somebody with established respiratory failure, high oxygen requirements, I think those same concerns would hold because it requires a bronchoscopic procedure.’

‘Well it’s bedside, it’s fast and it looks straightforward. All of those are advantages.’

‘The problem is, and the problem with the study, and the problem then with taking the technology forward to get people to use it, would be that if you add a layer of complexity to making your diagnosis…Doing the bronchoscopy and getting the lavage, that’s fine. Some units do that anyway, and we are toying with doing that routinely. But then adding on the layer of looking [at the bacteria]…You will get a result back from that within 48 hours. I guess the advantage of this technology is you get a result, as such, immediately [whether] bacteria or not, but of course it’s too easy to give two days of antibiotics. That will be the problem with getting people to use it. Because they would go, “Fantastic, we can do this test, but it was just easier to give two days of antibiotics.” And I know that’s not great’

‘…there are some antibiotics which have got much more potency against Gram-positive than Gram-negative organisms, and vice versa’.

‘…in the past year we had 800 ventilated patients, I think it was, and we had 15 ventilator-associated pneumonias. A bed in our intensive care unit costs £2,000 per night. So, if you can prevent 15 ventilator-associated pneumonias that might delay somebody’s discharge for 2 or 3 days, that’s then 15 times that £2,000 times 3. So, that’s a lot of money.’

‘…one of the differentials of new infiltrates on your chest x-ray is lung injury and leaky lungs caused by inflammation, rather than infection. So, if somebody is deteriorating it could be because they’ve got acute lung injury, rather than infection. So, if you can actually put that probe down and actually say, “Hang on. There’s lots of bacteria in there, this is infection.’

‘If we’re coming back to the question that I said about knowing what a VAP is, if it’s the case that seeing bacteria in the alveoli tells you that it’s definitely a VAP, and not seeing bacteria in the alveoli tells you it’s definitely not a VAP, then that would push a lot of people into doing it. If that’s the gold standard test and it’s not too invasive and not too difficult, you might say, “Well look, we can stop all this business of squirting saline into the lungs twice or three times a week. Save the cost of growing that stuff in the lab because it turns out it’s no good.” Instead, we’ll agree that on Monday and Thursday each intubated patient gets a bronchoscopic guided fluorescent test which might cost a couple of hundred quid for each patient on a Monday and a Thursday because we’re going to use a disposable bronchoscope. But maybe that saves on antibiotics because we find out that our antibiotic use goes down, so we’re not spending £50, £60 a day on tazocin or something. It would depend a bit on the definition of a VAP and how useful that technology turned out to be. If it was highly useful and had a high positive and negative predicted value, then I think you’d see clinicians saying, “Okay, I can do that.” In the same way that people will buy expensive echo machines and go to the trouble of spending half an hour a day echoing people because they believe that looking at the heart is important in ICU. Whereas that wasn’t something that was done before. Does that make sense?’

‘The bronchoscope does have advantage, in terms of there’s a dual procedure there, in terms of inspecting the airways, and you often come up with surprises; but actually, that’s not necessarily in terms of the diagnosis of VAP… You know, there may be practical

areas in terms that you may actually identify in terms of mucus plugging that you can actually manage with a lavage, and clear mucus plugs, etc… Well, yes. Yes. So, the bronchoscope… I suppose there's the potential of having a therapeutic component to the bronchoscopy, but obviously, that lengthens the procedure…'

'I like it because it falls in the category of explicit data for clinicians. You can actually see the bacteria, it's not like a test which is a surrogate marker. I think it will be more powerful because it's a direct reflection of actual biology, so it's more powerful than, say, procalcitonin. I would even say PCR, because you can actually see the bacteria. I think probably the most powerful effect would be if you had a patient where it was in balance, the decision was in balance as to whether to stop or start antibiotics and you saw the dramatic improvement in the pattern of apparent bacteria using the probes, then you're probably more likely to stop the antibiotics and even then use it for surveillance.'

'There's been lots of stuff over the years of imaging lungs, haven't there? And trying to use markers to show up bacteria. This is the first one I've seen where it's actually direct, rather than indirect. Normally it's indirect through scanning after injecting markers… Well, the indirect stuff's not caught on, and not influenced practice, has it? So, it's not been of any use really in clinical practice. It's all great in theory and in the lab. If you've got a pathological sample on the bench though, it's fine, but actually, I'm not so sure how useful they are; they've never been shown to be in vivo.'

'So I suppose if you have a chest X-ray with some specific area of opacification, then you're pretty happy that your probe is actually in that particular area, and you can't see any bacteria, well, maybe you could think it might rule it out.'

'I think it's interesting. I think it's a way of getting into the workplace. I think there might be spin-offs for this in respiratory medicine. I think the sort of patients they're looking at – you know, the chronic lung diseases, the cystic fibrotics, and people like that – they may be very useful in those patients, for routine screening, you know. I think there are lots of potential uses for this sort of technology, but we need to see how it works in practice.'

'I was going to say within the lung whether it would be useful for cytology for malignancy or something like that… very occasionally, if we're suspicious of malignancy, we tend to get our respiratory colleagues to do the bronchoscopies then. It did occur to me that this might be a technique that could be used for that. I'm asking a question rather than suggesting it, if you see what I mean?'

Theme 4: Barriers to adoption

‘although the technique is fairly straightforward, it involves using bronchoscopy, there will be a learning curve, so that, I would say, is the most problematic barrier to delivering the technology.’

‘So, I mean, you’d obviously have to have somebody who was reasonably skilled at bronchoscopy, or actually getting the probe into the segment of the lung. So, for example, if you had changes on chest x-rays, say, a change on the lower part of the left lung, you’d have to have somebody who had enough knowledge to actually get that probe into the left lung, and they weren’t doing it upside down or have the thing, the image inverted, so that, actually, they were putting the probe down into the wrong lung and actually saying, “There’s no bacteria lighting up here. Everything is fine.” So, that would be important, but that’s just a training issue.’

‘[On its possible implementation] I think there are so many things that would depend on. It would depend on, I suppose, the technical aspects of the procedure and how difficult it is. As I say, I’ve been told it’s not that difficult technically. I suppose that would encourage or put people off. Clearly, cost is always going to make a difference. If the smart probes… [these can be] extremely expensive as disposables then you’re going to use it more sparingly – when you really feel it would make a difference, as opposed to as almost like a screening test. Again, we do work in a system of limited resource and we have to direct that resource efficiently.’

‘…transpulmonary stuff at the moment in the UK tends to be done by respiratory physicians. There are not a lot of intensive care doctors that are just doing trans… you know, sticking probes and deliberately trying to perforate bronchi into lung tissue. So, that’s quite interesting as in the aspect is something which I don’t think many people have done, and I’m not sure how many would be comfortable with doing without training, because we’re used to doing endobronchial aspirations, not transbronchial, which this is essentially what it is.’

‘I suspect it would only be consultants who- our residents would be part of the team doing this technique, but I think it would be consultant-led intervention, I don’t think we would have junior doctors doing it.’

‘Anybody with severe [end of their lung] disease, whether that’s pneumonia, or ARDS, or whatever the underlying condition is, if they got a pneumothorax and lost a lung that could be fatal. So anything that puts them at risk of pneumothorax would be potentially difficult to undertake. The problem is that the patient who has got a deteriorating chest is exactly the sort of patient where you might want a better test for VAP, but they are the patient whose oxidation has already worsened, so they are much more vulnerable, both from the prolonged bronchoscopy but also from the transbronchial puncture.’

‘with any probe that is a relatively small diameter, I mean, I don’t know how rigid the end of the probe is but, I mean, pressure is force over area. So, if something with a very small diameter is going into the lungs, again, sending pressure then there is a risk of puncturing the probe into bits of the lung that you don’t want to go into. I would have thought the risk was fairly minimal but, again, I suppose you won’t really know that until you try this clinically… when somebody is unconscious and on a ventilator, and you’re doing that, then they’re not going to respond to you when you do that. So, theoretically, it is a risk of you sticking that probe, like, puncturing something that you shouldn’t puncture. The risk of that would be what is called a pneumothorax, if you get air into the pleural space, if you really went far too far with it but, I mean, you’d really have to be being pretty brutal to do that. So, to be honest, my gut instinct is that the risk is probably fairly low.’

‘You’ve still got to push it through and into the [alveolus]. I would be interested to know in a sick lung patient what your pneumothorax rate might be… even if the pneumothorax was very low the consequence of pneumothorax might be devastating. That’s the problem. Even if the rate was very low, if you kill a patient with it, for a diagnostic test, then you’ve suddenly offset any advantage you were going to gain. If it was done. That bit you can’t take away, I guess. Without the bronchoscope, there is some advantage in that. Because obviously the problem with a bronchoscope, even a small scope, is it blocks your tube while you're doing the investigation, and so ventilation is always impaired while you're doing the investigation. So the time that you do it. And obviously the sicker someone’s lungs are the quicker you’ve got to be. And then doing something fiddly is going to slow you down. So I guess if you didn’t have to do the bronchoscopy. The problem with not doing the bronchoscopy is of course you’ve absolutely no idea then where the catheter has ended up. So the [business] I talked about before of targeting a certain area of the lung, the left lower lobe or whatever it is, you couldn’t do that, because you don’t know whether the catheter has bounced and gone up into the right upper. You just don’t know where it’s ended up. We routinely do, like I say, blind alveolar lavage, which obviously we don’t know which side that’s gone down, but of course it’s hopefully not making a hole in the lungs.’

‘There’s the basic concern of using bronchoscopy in people you are critically oxygenating, although I think that’s not going to be an issue that often. I think there’s a concern about the cost because if we can’t do X because we’re using this thing five times a week then, you know, X might also be beneficial. Then, I suppose, it’s just a small [risk] about transbronchial rupture – whether there was any chance of causing things like pneumothoraces [pushing in too far] and whether there are any clinical risks to the patient. It hasn’t been something that I’ve heard of before but I would want to know about that.’

‘I would have concerns about a deliberate transbronchial puncture, to be honest… That it might be dangerous… it seems like it's a blind technique. And from the video it says you push until you get some more resistance and then push it through. And you don't know where you're pushing it through, or it doesn't look like you could see the damage that you might have caused in the bronchial wall… I would not go near this device at the moment, until somebody else has tested it.’

'…it would concern me because the current techniques that we use are BAL and so on, bronchoscopy, they don't require us to do a puncture. In fact, we would try not to cause a puncture. Deliberately causing one would be different for the experience of most intensivists. We might be a bit concerned that in the sickest of patients on highish ventilatory pressures, that that could be detrimental… [Risk?] Yes, because A. it would probably increase the risk of bleeding, and B. it would increase the risk of pneumothorax.'

'I note the question there about transbronchial puncture actually. So, obviously, I suppose things have to be worked out from a safety profile, as well… I think there's transbronchial puncture, and there's transbronchial puncture. From a respiratory background, we would have experience in using transbronchial biopsies, actually using biopsy forceps to take smaller alveolar type samples for diagnosing in particular, for example, interstitial lung disease. Now, obviously, with these procedures, when we do those procedures, they carry a risk of a pneumothorax. You know, a relatively small risk, but even if that is in the low percent, that is still a significant risk if you have a patient who is positively pressure ventilated. So, I suspect this probe, by the looks of it, as you said, is pretty small, and I suspect it will be quite innocent, but I would be interested to know what the pneumothorax risk is, because I think it would then be the safety aspects, especially if there is a potential in this sort of situation that this procedure is something that can be done in the same patient on more than one occasion.'

'…some people who have chronic obstructive airways disease, they can get large, dilated air sacs within their lung, called bullae. So, people with very bad COPD are possibly at a higher risks. Patients who are being inflated with very high airway pressure, possibly, as well. Again, anything you do in medicine is a risk-benefit, and it may be that if the risk, and I think the risks, probably, are fairly low and I suspect that the advantage of actually getting a bacteria result and targeting your antibiotic therapy probably outweigh what- I'm just talking off the top of my head, I'm just thinking out loud, but I think the risk is probably fairly low. Some patients who have got big, dilated bits of their lung, like, when you've got very bad emphysema you can get, as I say, these big bullae within your lungs. So, there is a theoretical risk of puncturing one of them if you get very bad COPD but, again, I think the risk is fairly low.'

'There are patients at risk of pneumothorax in general, so if you've got bad ARDS in your lungs, it's particularly [stiff] and you're having to use high pressures – which we try and avoid nowadays, to be honest – to ventilate them, you're more at risk of pneumothorax, I suppose. It's less the absolute risk of pneumothorax and more the relative risk of if you get a pneumothorax that would cause more problems. For example, if you're intubated for an overdose and you're unconscious and we're worried you've got a ventilator-associated pneumonia but you're still on 30% oxygen, you're 'safeish'. Then a pneumothorax might not be a big deal. If you're at 95% oxygen and we were doing this at a push because we really needed to know and suddenly you cause a pneumothorax then you're going to take that patient right over the edge.'

'[…are there patients that are especially vulnerable to pneumothorax?] Well, I suspect that depends on the underlying clinical presentation. If you've got patients who were, for example, with COPD and an emphysema pattern with bullous lung disease, they would be susceptible to pneumothoraces. Obviously, you have to take that into consideration, dependent on the airway pressures, the level of PEEP, that's being delivered to the lungs when you're ventilating the patient, because there's a positive respiratory pressure which may… if there is a tiny air leak that then is driving pressure out into the pleural space.'

'The commonest things are pre-existing COPD or emphysema effectively. Patients with pre-existing disease… There are certain pneumonias that are more likely to cause cavitating lung lesions. Again, they are at more risk of developing pneumothorax barotrauma from the ventilator.'

'anybody with severe sepsis, anybody with blood clotting abnormalities. I guess the worse end of the spectrum, so anybody who has had very high oxygen requirements already. But it seems to me it could potentially cause- Well, at the worst case a pneumothorax.'

'For example, if you put this technology into an ICU and the first patient [develops a] pneumothorax, then probably that's the end of that technology in that centre. They would never use it again.'

'if your diagnostic test even has a risk of killing one patient then people won't want to do it. And if the diagnostic test is too cumbersome or complicated then, again, people won't do it, because they will say, "Oh, we will do the lavage maybe, but we will just give some antibiotics for a couple of days and wait for the result." That's the problem really.'

'…well if you're doing multiple sites, you're increasing the risk.'

'…patients where you'd be getting most out of this diagnostic technology would be the patients who are more likely to develop a complication from trauma to the lung. For example, if you had a patient with high transpulmonary pressure with ARDS, adult respiratory stress syndrome, then they are more likely to get anaemia thorax or complications related to bronchoscopy per se, in derecruitment, for example. So it's the law of diminishing return. The more you want to use the technology, the less you can use the technology if it has a downside.'

'The only drawback I can see is that if you look at that people would say, "Oh, it looks like a bit of a faff." That's probably what some people would say because you have to do it using a scope, don't you?'

'Cost isn't the most important thing, it's the actual human factor thing again. Can he be bothered to actually do it? If you're fairly clear, for example, you might start protocolise antibiotics, the patient gets better, you're not going to do this technique, probably.'

'[On using ETAs over bronchoscopic procedures] That was more to do with the time and effort required versus the benefit you actually get, which is relatively small.'

'Hopefully you will have done a chest X-ray by this point, and there may be an area of interest, so a targeted location. If it's left lower lobe then obviously you will want a sample from the left lower lobe. But then whether you do representative samples from the upper

and the right on the other side I don't know. It’s difficult, because even if you're in an infected lobe you might have just gone down the wrong segment, and there’s no way from the inside of knowing. It’s relatively blind where it’s going beyond that. So you’ve no idea whether you're going to hit the right segment.’

‘That could be a positive and a negative. If you said to me, “The good news is you can hunt around the lung looking for it,” then I might be a bit naturally suspicious and say, “Are you telling me that I have to be really careful where I put it or I’m going to miss a VAP?”… Whereas if you could tell me that I can stick it just about anywhere and it will tell me if there’s a VAP, then that’s much more powerful from an intensivist point of view.’

‘it comes down to evidence base, so the evidence base of two things, really, doesn't it? One, whether this actually makes patients get better. It would be nice to think that could be demonstrated in a trial. The second thing would be whatever the presence of bacteria actually means, because pretty much I think every ventilated patient has got some bacteria down there. And the question is going to be is that causing infection or not?... And then how would that then be translated to whether this is colonisation or infection? Because presumably we'd have a protocol where we can see other signs of actual- This being an infection, as you know, clinical signs, etc., of the patient, but then presumably are there any analyses of any cytokines or anything in the alveolar fluid which then would fit together with the visualisation of the bacteria? If that makes sense.’

‘I think I'd have to just say potentially [alter clinical decision making] yes, because I think it's still- Because I don't know if you put this technology down 100 patients on a ventilator, whether you would see bacteria in 100 patients.’

‘What obviously you can’t tell from that is what the resistance pattern might be… but you would still want your microbiology data for the resistance pattern… You might say, “Well, it’s a gram-positive,” but is that staph aureus or MRSA? Is it going to be resistant to your normal treatments or not?... knowing there’s bacteria down there helps you, but doesn’t help you massively, in terms of your antimicrobial choice, I guess.’

‘…they’ve got to demonstrate that it gives added benefit because, you know, it’s not going to be cheap. It’s a procedure. Does it give added benefit over our empirical pathway at the moment, of managing it? Because, as I said, all we seem to be getting is whether it’s gram-negative or gram-positive. So, all you’re going to do is then, if it’s both, you’re going to give broad-spectrum antibiotics on both. There are only gram-negative or gram-positive; you’re going to go one, or the other. But it doesn’t actually help you, at this stage, as far as I can see, then look at being much more targeted treatment. If you don’t have the actual bacteria, and you don’t have culture to culture and sensitivity. I’m yet to be convinced that the micro aspirations are going to be helpful. But I may be wrong.’

‘In my view, it’s difficult to get stuff up a bronchoscope looming sometimes, let alone [OMA aspiration]… You’re much more likely to be able to pull the probe out and cut the end of it off and send that off. You’re much more likely to get that, because the contaminant is on the probe itself.’

‘Also, that you're not putting them at increased risk of the probe sitting there itself creating infection, which would be the other potential, because you've actually- It's no worse than doing physiotherapy in a way, but you are leaving [something] there, leaving a foreign body in the alveolus, which potentially could act as a source of-Focus for infection to develop.’

Theme 5: Evidence requirements

'In my view, it's difficult to get stuff up a bronchoscope looming sometimes, let alone [OMA aspiration]… You're much more likely to be able to pull the probe out and cut the end of it off and send that off. You're much more likely to get that, because the contaminant is on the probe itself.'

'Also, that you're not putting them at increased risk of the probe sitting there itself creating infection, which would be the other potential, because you've actually- It's no worse than doing physiotherapy in a way, but you are leaving [something] there, leaving a foreign body in the alveolus, which potentially could act as a source of- Focus for infection to develop.'

'…if it is a technique that you're actually going to make a change in antibiotic therapy, or something, then you probably want high sensitivity and a high specificity, actually.'

'I think, clearly, we've mentioned around the safety aspects. In terms of efficacy, I think there would need to be a comparison with the current Gold Standards that we have, and that would be ideally against a bronchoscopic lavage in terms of the sensitivity and specificity of the results of the organisms.'

'Well if I did that, say I was doing that on a patient and if it was completely negative, then it would influence my decision to not treat for gram-negative infection. If it was heavily positive, with a lot of growth like that, then it would obviously influence my decision to treat for a gram-negative infection. Obviously, I'd want to know what the sensitivity and specificity were before I could tell you whether it'd make a big difference or not.'

'it had a high positive predicted value for VAP, it had a high negative predicted value for VAP. I think if it had those things and it was safe, then actually by definition it would almost inevitably be cost-effective because it would mean that you'd save on pointless antibiotics. You'd save on missed VAP. The cost-effective thing will probably naturally follow from it if it works.

'The technology is going to come with a price tag and so people are going to want – probably want to say that there's superiority, rather than just non-inferiority.

'I would have thought the first study would be an observational study of the meaning of seeing the bacteria in there, and then logically deciding whether that's an infection or not… also that could be a safety study so that you can rule out the actual technology itself causing a problem.'

'the goal standard would have to be a BAL.'

'I would say a BAL would be the best comparator because in terms of- Well, for one thing, in terms of selecting patients for a clinical trial, your selection criteria would have to be those patients in whom you would have done a BAL anyway.'

'I mean… we almost exclusively use endotracheal aspirates. I suppose if you compared to BAL it would be less valid for us, personally. That doesn't mean it would be invalid. In theory, if you're showing improved accuracy versus BAL and we know that BAL is more accurate, the assumption would be it's better than what we're doing. The clinical endpoint would probably need to be more patient-centred than simply better accuracy of what bacteria it was. You need to show some sort of clinical benefit, be that ventilator length of stay or obviously mortality would be great, or something like that. Simply saying, "Better accuracy of antibiotics but then it doesn't affect anything else," then what you're doing is spending quite a bit of time each day doing this procedure that could be spent doing other things that might actually affect mortality or quality of life or the things that patients care about.'

'If you're thinking about a gold standard and the way of doing a trial, I think the practical problem that would be encountered is that lots of different units will be doing things differently, so practice will vary. Some places will be doing surveillance m-BALs. Some people will be doing direct broncs. Probably if you take it to our unit and said, "We'd like to trial this device." Then probably our unit would say, "Okay, well we'll do that, but we'll have to trial it against what our usual care is." Which is surveillance m-BAL and clinical decision making. If you say you want us to compare bronchoscopically guided BAL to this, we won't do that because we don't normally do that other thing. You'd probably have to do a pragmatic study of units doing what they normally do against using this technology. Otherwise they wouldn't want to do it and also, when you were trying to generalise the study, if people would look at it and go, "Well this study compared this technology to something we don't do, so I don't know what that means for me.".

'I think that's one of those where you have to really do it that way because too many people say, "We compared this new thing to something, to this." The commonest objection from the intensive care units is, "Yes, but we don't do that. I can't make a sensible decision about whether I should do this new thing, because we don't' do the thing that you've compared it to." The consultant body here – if anybody wants to do a study here; we do lots of studies – they say, "Well, that's fine. We're happy to do something new, but we're only going to compare that to the normal practice for our patients.".'

'…an effectiveness trial is important. In the end, you have to put this technology into target intensive care units and you'd have to make a decision as to whether you're going to put them into lung injury centres or whether you're going to put them everywhere, and just basically randomise the patients to this intervention versus standard care and see if you can measure treatment effect size that was clinically or statistically effective.'

'I think it's difficult to answer that because it's such a new technology, that without testing it- you obviously have efficacy but whether it is effective in terms of decision making, I think you just don't know until you put it into the real world.'

'I think a BAL would be your best bet as a gold standard. You would have to have some way of demonstrating better patient outcomes. Increasingly, if you look at critical care studies that are published, you're looking right out to 90 days at least. Crudely what I would say is, it may make a difference

with antibiotics, but if at 90 days the mortality is exactly the same then what's the point? I'm putting it a bit crudely, obviously, but that's the gist of it.'

'So, patients' outcomes are… I mean, the problem… is measuring patient outcomes in these sorts of trials in critical care is very difficult. What are you looking at? You're looking at mortality; a pretty crude blunt instrument, and affected by numerous other factors beyond infection. You're looking at morbidity; again, a lot of it is very subjective. You could look at more objective things like time to ventilation; length of stay in ICU, length of stay in hospital, long-term morbidity and mortality. So, where are we going? We can look at other things that are completely unrelated, like development of multiresistant bacteria… So, it depends what benefits you're looking at. Are you looking at societal benefits? Are you looking at cost benefits? Are you looking at patient benefits? I suspect that any trial doing this will probably be looking at focusing on… there will always be a mortality aspect because that's easy. That's unitary… There's stuff like length of ventilation, length of device use, hospital stay, is quite easy to measure. The more difficult stuff to measure is clinical cure... but clinical cure is meaningless in this context, particularly as most of the patients have chronic morbidities and mortalities… So, what are you curing them of; and actually, what is clinical cure? So, it might be a complete resolution of chest X-ray changes, but that will be at three months, or six months. You know, it's not going to happen in a week. So, it is quite difficult.'

'There are two aspects of cost, I suppose, aren't there? There's the broader societal cost to giving too many antibiotics, and I appreciate that. But the big problem… is antibiotics are so cheap that any diagnostic investigation to cause a cost savings, in terms of antibiotics, has got to be really, really cheap. That's one of the problems, I think. If your diagnostic test costs £100 and two days of antibiotics is £2.50 then people will just give antibiotics… [Also] procalcitonin, which we haven't touched on, but procalcitonin is a useful add-on investigation. Originally the company were saying it was £10 a test. I said, "When a day's antibiotics costs me £2, why would I do a £10 test?"'

' [Is cost a consideration?]Absolutely. It's just a nightmare now. We would have to make a business case. We would have to explain what added value it brought… most published studies how would have some sort of cost analysis anyway. Some of the information is usually there to start with. It's pretty difficult at the moment to get money for anything. It's an effort… if you use this technique and you show there's no infection, and you're saving the cost of antibiotics. If the evidence suggests that that happens quite a lot, then it would pay for itself.'

'You have to go down the channel of a scope. They might say, "Okay, well that's fine," but if we're in a unit where we don't usually bronc people then you've got to provide some really hard evidence that going to the trouble of doing a bronc- because they might say, "Well, okay, we've decided we're not going to do bronchoscopically guided BALs routinely and we're just doing non-bronchoscopically guided ones. We've not got the technology where we're having to do it bronchoscopically guided. Is this definitely superior to just doing a bronchoscopically guided BAL?" Maybe. What are the cost implications of then using a disposable scope each time? Using a non-disposable scope each time? How frequently would it be proposed that you'd do this? I think they're the kind of objections that you'd probably meet from sceptics, I would have thought.'

'The company will argue about cost savings, about early diagnosis and appropriate targeting of antibiotics that might improve the outcomes. But you'd want to see evidence of that. So to get it past NHS commissioners, I think they would want to be convinced that there were real potential advantages, because I can't imagine it's cheap.'

'I think it would require a little bit of training in units that do less BAL because it's easy enough working out which is left and which is right bronchi. Once you get to sub-segmental it is something that probably requires a little bit of training. I don't think it's in any way insurmountable. I think it's a skill that doesn't take that long to get the basics of. It might require a bit of practice but I don't think it's a massive barrier.'

'The other thing is, when you've got people who develop a technique, they get to be very good at it very quickly and they tend not to get any complications. When you let something out there in the wild as it were, that's when you tend to see your complications.'

'Yes. I mean I'd want to know what the risks were of that particular technique and device before I could say whether it would be useful to keep doing it. Also, what the evidence was in repeating it, if you see what I mean?'

'If they had a self-guiding probe yes, that would really just – I mean there are self-guiding double lumen tubes and things… that have got cameras on the tips of them. If you had a technology where it was all built in and it was pushed down an endotracheal tube or something for instance, then I think you'd find people saying, "Okay, that would be more attractive." It would feel less like you were making a major overhaul to the intervention. You're sticking something smart down the ET tube, rather than sticking something dumb like a suction catheter down it. That would make it more attractive.'

Appendix 4 – Guideline review

National and international guidelines were identified from web search, but also pointed out by clinicians themselves in the interactions with members of the team. National guidelines were reviewed to examine the care pathway for suspected adult VAP patients in the ICU.

Local guidelines for VAP were obtained by contacting clinicians via the UK Critical Care Specialty Group (UKCCRG). The request was disseminated by the co-applicant on the project and member of the group through their mailing list in October 2017. By approaching a national organisation, we aimed to achieve good national coverage. A data extraction form was used to retrieve information from local guidelines provided by different NHS hospitals across the UK. When extracting information, we reviewed whether VAP, CAP or antibiotics-related local guidelines were available within each hospital. We also considered whether the local guidelines were in line with which national or international guidelines. Due to absence of NICE guidelines for VAP, other guidelines were considered. These guidelines were proposed by different institutions and regulatory bodies, such as:

- British Thoracic Society (BTS)
- British Society for Antimicrobial Chemotherapy (BSAC)
- American Thoracic Society (ATS)
- Infectious Diseases Society of America (IDSA)
- Centers for Disease Control and Prevention (CDC)
- Canadian Thoracic Society (CTS)
- Association of Medical Microbiology and Infectious Disease Canada (AMMI Canada)
- Hospitals in Europe Link for Infection Control through Surveillance (HELICS) or European surveillance of healthcare-associated infections in intensive care units HAI-Net ICU (updated HELICS)
- European Respiratory Society (ERS)
- European Society of Intensive Care Medicine (ESICM)
- European Society of Clinical Microbiology and Infectious Diseases (ESCMID).

In total, we reviewed 16 local CAP/VAP/HAP guidelines from hospitals across the UK NHS, 10 of them had also local guidelines for VAP available or followed specific national guidelines.

Table 1: Characteristics of the 16 NHS England Trusts whose local guidelines were requested, ordered alphabetically by a commissioning region

	NHS Trust	Commissioning Region	Beds in the ICU (adults)	Guidelines used
**1**	Guy's and St Thomas' NHS Foundation Trust	London	82	local guideline for the use of empirical antibiotics
**2**	Royal Free London NHS Foundation Trust	London	85	
**3**	West Suffolk NHS Foundation Trust	Midlands And East Of England	9	local guidelines for all infections
**4**	James Paget University Hospitals NHS Foundation Trust	Midlands And East Of England	12	
**5**	Nottingham University Hospitals NHS Trust	Midlands And East Of England	103	

**6**	University Hospitals Birmingham NHS Foundation Trust*	Midlands And East Of England	71	BTS
**7**	South Tees Hospitals NHS Foundation Trust	North Of England	69	
**8**	University Hospitals Of Morecambe Bay NHS Foundation Trust*	North Of England	14	local guidelines for VAP
**9**	Leeds Teaching Hospitals NHS Trust	North Of England	106	
**10**	Royal Liverpool and Broadgreen University Hospitals NHS Trust	North Of England	37	local guidelines for VAP
**11**	The Newcastle Upon Tyne Hospitals NHS Foundation Trust	North Of England	86	Local guidelines for the Antimicrobial Treatment of Respiratory Infections in Adults
**12**	Royal Berkshire NHS Foundation Trust	South Of England	13	HELICS
**13**	Royal Cornwall Hospitals NHS Trust	South Of England	15	
**14**	University Hospital Southampton NHS Foundation Trust	South Of England	106	
**15**	Royal Gwent Hospital	Aneurin Bevan University Health Board, Wales	15	BTS (IDSA/ATS)
**16**	Dumfries & Galloway Royal Infirmary	NHS Scotland Dumfries and Galloway	20	HAI surveillance of HELICS criteria

Key: * local guidelines provided by 2 hospitals within the same trust.

Source: Critical Care Bed Capacity and Urgent Operations Cancelled 2017-18 Data, NHS Beds by organisation and specialty since 2017-18 , Annual trends in available beds by NHS Board of treatment and hospital, 2017/18.

Using methodology presented in Bray et al, 2020 [4], we extracted information from the guidelines which covered clinical, radiological and microbiological diagnostic criteria for VAP. When clinicians reported that VAP guidelines are available within their Trust we retrieved the information from those, else we looked at the specific national guidelines that were explicitly reported.

Table 2: Diagnostic criteria for VAP across hospitals in the UK

Diagnosis		*1*	*3*	*6*	*8*	*10*	*11*	*12*	*13*	*15*	*16*
Clinical diagnosis	Age ≥ 65	✓	✓	✓	✓	✓	✓	✓	✓	✓	✓
	Respiratory rate	✓	✓	✓	✓	✓	✓	✓	✓	✓	✓
	Blood pressure	✓	✓	✓	✓	✓	✓	✓	✓	✓	✓
	Mental status	✓	✓	✓	✓	✓	✓	✓	✓	✓	✓
	Temperature (Fever>38 or <35)	✓	✓	✓	✓	✓	✓	✓	✓	✓	✓
	New onset of purulent sputum	✓		✓	✓		✓	✓	✓	✓	✓
	Change in character of sputum	✓		✓	✓		✓	✓	✓	✓	✓
	Cough, dyspnoea, or tachypnoea	✓		✓	✓		✓	✓	✓	✓	✓
	Suggestive auscultation,	✓		✓	✓		✓		✓	✓	✓

	rhonchi, wheezing										
	Worsening oxygenation (O2)	✓	✓	✓	✓	✓	✓	✓	✓	✓	✓
	Oxygenation saturation<br>Pulse oximetry (PaO2/FiO2)	✓		✓	✓			✓		✓	✓
	Liver function tests			✓				✓			
	White Cell Count (WCC)		✓	✓	✓			✓	✓	✓	✓
	Sputum Gram stain			✓	✓			✓			
Radiologic diagnosis	Initial Chest X-Ray:<br>Pulmonary radiography	✓	✓	✓	✓	✓	✓	✓	✓	✓	✓
	tomogram (position of the tube)										
	New and persistent (>48-h) infiltrate<br>on chest radiograph	✓	✓	✓	✓	✓	✓	✓	✓	✓	✓
Microbiological diagnosis	Broncho-alveolar lavage (BAL)	✓		✓		✓	✓	✓	✓	✓	✓
	Non-bronchoscopic -alveolar lavage (N-BAL)							✓		✓	✓
	Protected specimen brush (PSB)	✓		✓			✓	✓	✓	✓	✓
	Distal protected aspirate (DPA)	✓		✓			✓		✓	✓	✓
	Quantitative culture of LRT specimen<br>(e.g., endotracheal aspirate)	✓		✓	✓		✓	✓	✓	✓	✓
	Alternative microbiology methods							✓		✓	
	Arterial blood gases			✓						✓	
	C-reactive protein (CRP)	✓	✓	✓	✓	✓	✓	✓	✓	✓	✓
	Urea nitrogen	✓	✓	✓	✓	✓	✓	✓	✓	✓	✓
	Blood cultures	✓	✓	✓	✓	✓	✓	✓	✓	✓	✓
	Sputum cultures	✓	✓	✓	✓	✓	✓	✓	✓	✓	✓
	Urine (legionella, pneumococcal)	✓	✓	✓	✓	✓	✓	✓	✓	✓	✓
Included # by each Trust		22	14	26	21	14	21	25	22	26	24

✓ Retrieved from the international and national guidelines

✓ Retrieved from the local guidelines and mails from clinicians

Local guidelines for VAP in adults are rarely available within the trusts/hospitals across UK. They are mainly covered by guidelines for antibiotic usage, respiratory infections, ICU or national guidelines. There is a lot of variability within the scoring systems used across UK. In this exercise we converted the reported scoring systems to specific criteria used as a component to derive the score. For example, if the guidelines reported that *CURB 65* score is used, then we would tick the age ≥ 65, respiratory rate, blood pressure, mental status and urea nitrogen.

When same diagnostic criteria was included in local and national guidelines, the national guidelines would have a precedence (thus the orange colour code would prevail). In two cases we received local guidelines from the same Trust, but different hospital. In that case, we selected all the criteria covered by both guidelines.

In a recent exercise, an international, multidisciplinary panel of experts reviewed and ranked diagnostic tools used for the diagnosis of VAP in clinical practice. They agreed upon the following list of diagnostic criteria: chest X-Ray, blood cultures, endotracheal aspirates, mini-BAL, CRP/PCT and Gram stain[5]. Apart from Gram stain, these are also included in the diagnostic criteria for VAP across all hospitals we retrieved guidelines from NHS UK. Gram stain which was present in one of four reviewed local guidelines for VAP.

Among the invasive techniques, transbronchial biopsy, was ranked riskier followed by BAL. UK hospitals that provided us with local guidelines for VAP all suggest the use of BAL as a diagnostic to confirm VAP. In the absence of gold standard for the diagnosis of VAP, BAL is seen as current best standard recommended by European guidelines as opposed to non-invasive and semi-quantitative sampling recommended by American guidelines[6].

According to a 2014 national survey across UK Intensive Care Society[7], around 60% of clinicians comply in their management and practice with the Canadian Thoracic Society guidelines, 55% with the American Thoracic Society guidelines, 49% use HELICS (Hospitals in Europe for Infection Control through Surveillance) criteria and 28% comply with Guidelines from the British Society of Antimicrobial Therapy, specific radiological and clinical criteria for the diagnosis of VAP.

Appendix 5 – COREQ Checklist

We referred to the COREQ checklist to ensure appropriate and comprehensive reporting [8].

No	Item	Guide questions/description	Lines
Domain 1: Research team and reflexivity			
Personal Characteristics			
1	Interviewer/facilitator	Which author/s conducted the interview or focus group?	132-151
2	Credentials	What were the researcher's credentials? E.g. PhD, MD	132-151, 161-163
3	Occupation	What was their occupation at the time of the study?	132-151
4	Gender	Was the researcher male or female?	Not relevant
Relationship with participants			
5	Experience and training	What experience or training did the researcher have?	132-151, 161-163
6	Relationship established	Was a relationship established prior to study commencement?	N/A
7	Participant knowledge of the interviewer	What did the participants know about the researcher? e.g. personal goals, reasons for doing the research	See Appendix 1
8	Interviewer characteristics	What characteristics were reported about the interviewer/facilitator? e.g. Bias, assumptions, reasons and interests in the research topic	See Appendix 1
Domain 2: Study design			
Theoretical framework			
9	Methodological orientation and Theory	What methodological orientation was stated to underpin the study?	159-163
Participant selection			
10	Sampling	How were participants selected? e.g. purposive, convenience, consecutive, snowball	138-144
11	Method of approach	How were participants approached? e.g. face-to-face, telephone, mail, email	138-144
12	Sample size	How many participants were in the study?	138-144
13	Non-participation	How many people refused to participate or dropped out? Reasons?	143-144
Setting			
14	Setting of data collection	Where was the data collected? e.g. home, clinic, workplace	135-136
15	Presence of non-participants	Was anyone else present besides the participants and researchers?	N/A
16	Description of sample	What are the important characteristics of the sample? e.g. demographic data, date	141-144
Data collection			
17	Interview guide	Were questions, prompts, guides provided by the authors? Was it pilot tested?	132-151
18	Repeat interviews	Were repeat interviews carried out? If yes, how many?	N/A
19	Audio/visual recording	Did the research use audio or visual recording to collect the data?	135-136
20	Field notes	Were field notes made during and/or after the interview or focus group?	N/A
21	Duration	What was the duration of the interviews or focus group?	135
22	Data saturation	Was data saturation discussed?	145-146. 422-425
23	Transcripts returned	Were transcripts returned to participants for comment and/or correction?	Yes
Domain 3: analysis and findings			
Data analysis			
24	Number of data coders	How many data coders coded the data?	159-163
25	Description of the coding tree	Did authors provide a description of the coding tree?	N/A
26	Derivation of themes	Were themes identified in advance or derived from the data?	136-137
27	Software	What software, if applicable, was used to manage the data?	See Appendix 2
28	Participant checking	Did participants provide feedback on the findings?	No
Reporting			
29	Quotations presented	Were participant quotations presented to illustrate the themes / findings? Was each quotation identified? e.g. participant number	See Appendix 3
30	Data and findings consistent	Was there consistency between the data presented and the findings?	N/A
31	Clarity of major themes	Were major themes clearly presented in the findings?	See Results section
32	Clarity of minor themes	Is there a description of diverse cases or discussion of minor themes?	See Results Section

References

1. UK Transcription. 2019; Available from: https://www.uktranscription.com/.
2. Chandra, Y. and L. Shang, *An RQDA-based constructivist methodology for qualitative research.* Qualitative Market Research: An International Journal, 2017.
3. R Core Team, *A language and environment for statistical computing.* R Foundation for Statistical Computing, 2018.
4. Bray, A., et al., *High Variability in Sepsis Guidelines in UK: Why Does It Matter?* Int J Environ Res Public Health, 2020. **17**(6).
5. Ferreira-Coimbra, J., et al., *Ventilator-associated pneumonia diagnosis: a prioritization exercise based on multi-criteria decision analysis.* Eur J Clin Microbiol Infect Dis, 2020. **39**(2): p. 281-286.
6. Torres, A., et al., *International ERS/ESICM/ESCMID/ALAT guidelines for the management of hospital-acquired pneumonia and ventilator-associated pneumonia: Guidelines for the management of hospital-acquired pneumonia (HAP)/ventilator-associated pneumonia (VAP) of the European Respiratory Society (ERS), European Society of Intensive Care Medicine (ESICM), European Society of Clinical Microbiology and Infectious Diseases (ESCMID) and Asociación Latinoamericana del Tórax (ALAT).* Eur Respir J, 2017. **50**(3).
7. Browne, E., et al., *A national survey of the diagnosis and management of suspected ventilator-associated pneumonia.* BMJ Open Respir Res, 2014. **1**(1): p. e000066.
8. Tong, A., P. Sainsbury, and J. Craig, *Consolidated criteria for reporting qualitative research (COREQ): a 32-item checklist for interviews and focus groups.* International Journal for Quality in Health Care, 2007. **19**(6): p. 349-357.
